# The molecular and metabolic program by which white adipocytes adapt to cool physiologic temperatures

**DOI:** 10.1371/journal.pbio.3000988

**Published:** 2021-05-12

**Authors:** Hiroyuki Mori, Colleen E. Dugan, Akira Nishii, Ameena Benchamana, Ziru Li, Thomas S. Cadenhead, Arun K. Das, Charles R. Evans, Katherine A. Overmyer, Steven M. Romanelli, Sydney K. Peterson, Devika P. Bagchi, Callie A. Corsa, Julie Hardij, Brian S. Learman, Mahmoud El Azzouny, Joshua J. Coon, Ken Inoki, Ormond A. MacDougald

**Affiliations:** 1 Department of Molecular & Integrative Physiology, University of Michigan Medical School, Ann Arbor, Michigan, United States of America; 2 Department of Internal Medicine, University of Michigan Medical School, Ann Arbor, Michigan, United States of America; 3 Morgridge Institute for Research, Madison, Wisconsin, United States of America; 4 National Center for Quantitative Biology of Complex Systems, Madison, Wisconsin, United States of America; 5 Agilent Technologies, Inc., Santa Clara, California, United States of America; 6 Department of Biomolecular Chemistry, University of Wisconsin, Madison, Wisconsin, United States of America; 7 Department of Chemistry, University of Wisconsin, Madison, Wisconsin, United States of America; 8 Life Sciences Institute, University of Michigan, Ann Arbor, Michigan, United States of America; Duke University, UNITED STATES

## Abstract

Although visceral adipocytes located within the body’s central core are maintained at approximately 37°C, adipocytes within bone marrow, subcutaneous, and dermal depots are found primarily within the peripheral shell and generally exist at cooler temperatures. Responses of brown and beige/brite adipocytes to cold stress are well studied; however, comparatively little is known about mechanisms by which white adipocytes adapt to temperatures below 37°C. Here, we report that adaptation of cultured adipocytes to 31°C, the temperature at which distal marrow adipose tissues and subcutaneous adipose tissues often reside, increases anabolic and catabolic lipid metabolism, and elevates oxygen consumption. Cool adipocytes rely less on glucose and more on pyruvate, glutamine, and, especially, fatty acids as energy sources. Exposure of cultured adipocytes and gluteal white adipose tissue (WAT) to cool temperatures activates a shared program of gene expression. Cool temperatures induce stearoyl-CoA desaturase-1 (SCD1) expression and monounsaturated lipid levels in cultured adipocytes and distal bone marrow adipose tissues (BMATs), and SCD1 activity is required for acquisition of maximal oxygen consumption at 31°C.

## Abstract 2

Adipocytes in bone marrow, subcutaneous and dermal sites generally exist at temperatures below 37°C. This study identifies the molecular and metabolic program that adapts white adipocytes to these cooler environments.

## Introduction

Survival of euthermic animals is dependent on tight regulation of body temperature and cellular function in environmental conditions below thermoneutrality. Although mammals have developed a complex system to defend core body temperature within a narrow range, the exterior and extremities remain much cooler. Whereas the body core is maintained at a relatively constant approximately 37°C across a broad range of environmental conditions, the body shell, which includes peripheral extremities and subcutaneous regions of the trunk, is characterized by a wider spectrum of temperatures. For almost a century, scientists have known that temperature gradients exist across the body, from central to distal, and superficial to deeper regions [1]. For example, subcutaneous tissues in the human calf are generally approximately 32°C but can dip to below 30°C in the cold and increase to 37°C when it is hot or during exercise [1,2]. Even subcutaneous tissues of the chest and back are generally 2°C below core temperature and function in a more dynamic range (32 to 37°C) than cells located deeper within the body [2].

Temperature is detected by sensory neurons within skin, core, and brain, which transmit signals to the central thermoregulatory network. After signal integration, specific hypothalamic neurons regulate body temperature through a combination of behavioral and physiological mechanisms [3,4]. Physiological responses to cold include vasoconstriction within skin to limit cooling, as well as shivering and adaptive thermogenesis to generate heat. The canonical adaptive thermogenesis pathway involves activation of sympathetic drive to increase brown adipose tissue metabolism and UCP1-dependent uncoupling of the mitochondrial proton gradient from ATP synthesis [5,6]. Activation of adaptive thermogenesis may be an intrinsic cellular response in some contexts, for example, moving 3T3-F442A adipocytes from 37°C to cool temperatures (27 to 33°C) rapidly induces expression of UCP1 and uncoupled oxygen consumption [7]. Recent work has also uncovered noncanonical mechanisms for heat generation in beige/brite adipocytes, including a creatine kinase futile cycle [8,9] and ATP-dependent calcium cycling mediated by SERCA2b [10,11]. Whereas the response of brown and beige/brite adipocytes to cold stress has been extensively studied, comparatively little is known about the program by which white adipocytes adapt to cool temperatures.

White adipocytes are distributed throughout the body in discrete depots and the intrinsic cellular and metabolic properties of different populations are shaped by the specific niches in which they reside [12,13]. Whereas visceral white adipocytes are found within the body’s core, other populations, including subcutaneous, marrow, and dermal adipocytes, primarily exist in temperatures well below 37°C. Despite this, studies within the field have largely neglected environmental temperature as a variable when considering the molecular and functional characteristics of white adipocytes. Although exposure of white adipocytes to cooler temperatures does not induce known adaptive thermogenesis pathways, hints from historical literature suggest that cooler adipose tissue temperatures are associated with greater lipid unsaturation [14]. Tavassoli and colleagues and our own work have found similar correlations in rabbits, rodents, and humans, with distal marrow adipocytes containing a larger proportion of unsaturated lipids and proximal sites [15,16]. How cool temperature-adapted cells, such as subcutaneous and distal marrow adipocytes, are functionally different from warmer counterparts is unknown.

To explore the adaptation of white adipocytes to changing environmental temperature, we exposed cultured adipocytes to a “cool” temperature of 31°C, the temperature at which distal marrow and subcutaneous adipose tissues exist when animals and humans are at a room temperature of 22°C [1,2,17]. We found that exposure of cultured adipocytes and adipose tissues to cool temperatures induces expression of stearoyl-CoA desaturase-1 (SCD1), increases proportion of monounsaturated lipids within triacylglycerols (TAGs), and regulates expression of a shared set of transcripts. SCD1 is highly expressed in adipocytes and converts saturated lipids such as palmitoyl-CoA to monounsaturated lipids such as palmitoleic acid. Culturing adipocytes at 31°C increases anabolic and catabolic lipid metabolism and elevates oxygen consumption, which is largely fueled through use of pyruvate, glutamine, and, especially, fatty acids as energy sources. In addition to expansion of mitochondria and peroxisomes, we observed up-regulation of proteins involved in β-oxidation and complexes I, II, and III of the electron transport chain. Finally, SCD1 activity is required for the increase in maximal oxygen consumption and spare respiratory capacity observed during cool adaptation. Thus, the molecular and metabolic program for adaptation of white adipocytes to cooler temperatures is cell autonomous and distinct from canonical thermogenesis.

## Results

### Cool environmental temperatures induce SCD1 expression and lipid desaturation

**Fig 1:**
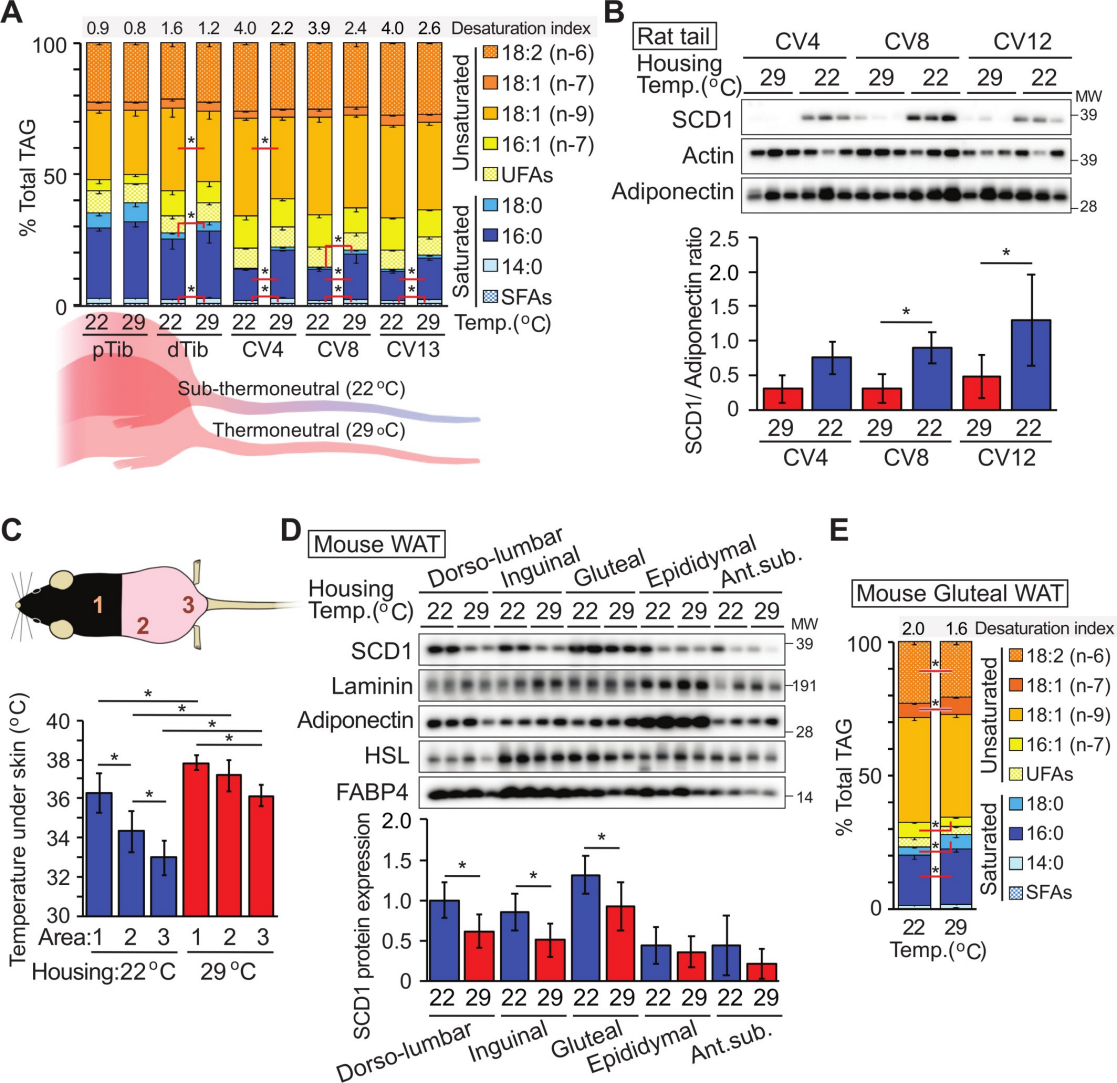
Lipid unsaturation and SCD1 expression are induced by cool environmental temperatures. (**A, B**) Rats were housed from birth to 11 weeks of age at standard room temperature (22°C) or thermoneutrality (29°C). (**A**) Increased proportion of unsaturated lipid in BMAT TAG of dTib and CV of rats housed at 22°C. Lipid composition of TAG for pTib and dTib and indicated CV were determined by GC (*n* = 6 except CV4 and CV13; *n* = 3). Desaturation index (16:1 + 18:1)/(16:0 + 18:0) is shown at top of graph. (**B**) Expression of SCD1 is elevated in CV from animals housed at 22°C compared to 29°C. SCD1 protein levels were normalized to adiponectin (*n* = 7–9). (**C**–**E**) Mice were housed from birth to 13 weeks of age at 22°C or 29°C. After weaning, posterior hair was removed weekly. (**C**) The subdermal temperature of mice housed at each temperature (*n* = 7, 8). (**D**) Elevated SCD1 expression in subcutaneous WAT depots of mice at 22°C. SCD1 protein expression was normalized to geometric mean value of the following proteins; adiponectin and laminin (shown), as well as perilipin, and HSP70 (*n* = 8 or 9). (**E**) Increased proportion of unsaturated lipid in TAG of gluteal WAT of mice housed at 22°C compared to 29°C (*n* = 5). Data are presented as mean ± SD. *p < 0.05. Uncropped western blots are provided in S1 Raw Images, and numerical data for all graphs are provided in S1 Data. BMAT, bone marrow adipose tissue; CV, caudal vertebra; dTib, distal tibia; FABP4, fatty acid-binding protein 4; GC, gas chromatography; HSL, hormone-sensitive lipase; pTib, proximal tibia; SCD1, stearoyl-CoA desaturase-1; SFAs, saturated fatty acids; TAG, triacylglycerol; UFAs, unsaturated fatty acids; WAT, white adipose tissue.

We previously reported that isolated bone marrow adipocytes (BMAds) from the distal tibia (dTib) of mice elevated *Scd1* expression and higher levels of monounsaturated lipids compared to those found in the proximal tibia or within white adipose depots [15]. We hypothesized that this increase in lipid desaturation occurs because distal BMAds exist on the cool end of temperature gradients present in rodents housed at 22°C. Indeed, in rodents housed at 22°C, temperatures across caudal vertebrae (CV) range from 38°C at CV1 and CV2 to 32°C by CV9 [17]. To test this hypothesis, we housed rats at room temperature (22°C) or thermoneutrality (29°C) from birth to 11 weeks of age. Lipidomic analyses revealed decreased levels of saturated fatty acids (myristic, palmitic, and stearic acids; shades of blue) and increased proportions of unsaturated fatty acids, particularly oleic acid, esterified within TAG isolated from distal bone marrow adipose tissue (BMAT) depots of rats housed at 22°C (Fig 1A). Interestingly, we did not detect temperature-dependent alterations in phospholipids (S1A Fig). We then focused our analyses on SCD1, an endoplasmic reticulum enzyme that catalyzes formation of monounsaturated fatty acids; specifically, it converts Coenzyme A-derivatives of myristic (C14:0), palmitic (C16:0), and stearic (C18:0) acids to myristoleic (C14:1, n-5), palmitoleic (C16:1, n-7), and oleic (C18:1, n-9) acids, respectively. Consistent with the lipidomic profile, SCD1 expression is also regulated by temperature, with considerably more SCD1 protein observed in CV and dTib BMAT depots of rats housed at 22°C compared to 29°C (Fig 1B).

To explore if changes in gene expression induced by cool temperatures are also observed in white adipose tissues (WATs), similar experiments were performed using mice exposed to cool environmental temperatures. In a subset of mice, posterior hair was removed to reduce insulation. In mice housed at 22°C, subcutaneous temperatures were found to be approximately 33 to 34°C, approximately 3°C lower than in mice housed at 29°C (Fig 1C). Consistent with this reduced temperature, SCD1 expression is higher in posterior subcutaneous depots (dorsolumbar, inguinal, and gluteal WAT) of mice housed at 22°C; in contrast, SCD1 is not altered in anterior subcutaneous or visceral WAT depots, where tissue temperatures are maintained at approximately 37°C (Fig 1D, S1B Fig). Expressions of laminin and adiponectin, as well as perilipin and HSP70, were not altered by environmental temperature and serve as loading controls (Fig 1D, S1C Fig). Hormone-sensitive lipase (HSL) and FABP4 appear to be more highly expressed at 22°C in inguinal and gluteal depots. Consistent with elevated SCD1, we observed decreased proportions of saturated fatty acids (palmitic and stearic acids) and increased palmitoleic and vaccenic acids within TAG of gluteal WAT of mice housed at 22°C (Fig 1E). These data indicate that monounsaturated lipid content is increased by exposure to cooler temperatures in both BMAT and posterior subcutaneous WAT.

### Cool temperatures increase SCD1 expression and lipid monounsaturation through a cell autonomous mechanism

**Fig 2:**
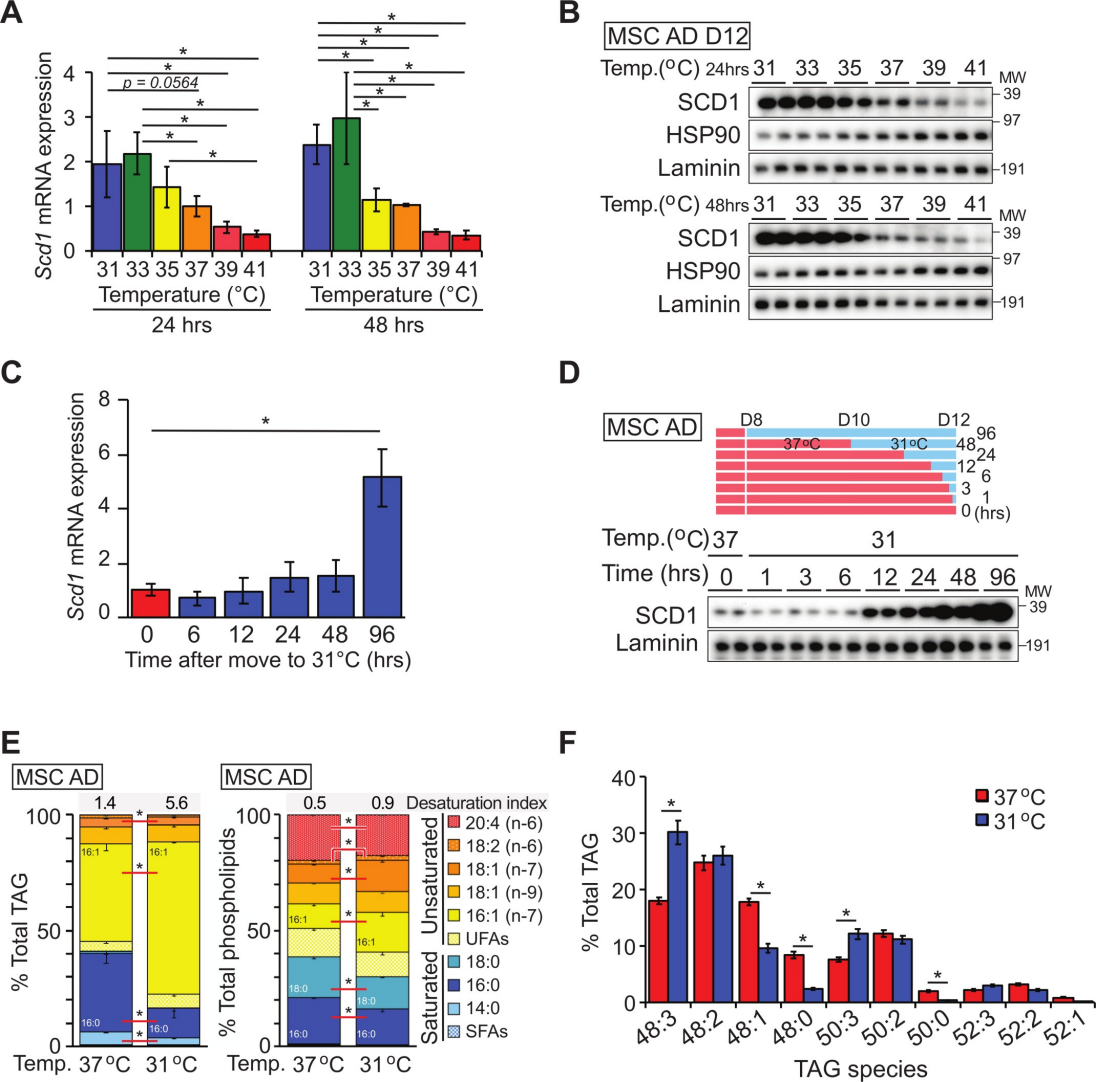
Adaptation of cultured adipocytes to cool temperatures induces expression of SCD1 and proportion of unsaturated lipids in TAG and phospholipid fractions. (**A, B**) Cultured adipocytes were incubated for 24 or 48 hours at indicated temperatures before collecting samples. Expression of SCD1 mRNA (**A**) and protein (**B**) were evaluated by qPCR and immunoblot, respectively. *Scd1* mRNA was normalized to the geometric mean value of *Hprt*, *Tbp*, *Gapdh*, *Rpl32*, *Rpl13a*, *B2m*, and *Rn18s* and is expressed as fold change relative to 37°C control (*n* = 4). (**C, D**) SCD1 mRNA (**C**) and protein (**D**) expression in adipocytes cultured at 31°C for indicated times. SCD1 mRNA was normalized to *Hprt* mRNA and is expressed as fold change relative to 0-hour control (*n* = 4). (**E**) Lipid composition of TAG and phospholipid fractions in adipocytes adapted to 31°C for 11 days (*n* = 6). Desaturation index at top of graph is (16:1 + 18:1)/(16:0 + 18:0). (**F**) TAG species in adipocytes adapted to 31°C for 11 days (*n* = 4). Data are presented as mean ± SD. *p < 0.05. Uncropped western blots are provided in S1 Raw Images, and numerical data for all graphs are provided in S2 Data. qPCR, quantitative polymerase chain reaction; SCD1, stearoyl-CoA desaturase-1; TAG, triacylglycerol.

To study the regulation of lipid desaturation and SCD1 expression by temperature more mechanistically, we established a cell culture system in which we could evaluate how white adipocytes adapt to cool temperatures. We chose to use mesenchymal stem cells (MSCs) because they can be isolated directly from transgenic mouse models of interest and are highly adipogenic [18]. A subpopulation of adipose tissues, such as subcutaneous WAT, distal BMAT, and dermal WAT, exist at temperatures as low as 31°C when animals are housed at 22°C [2,17]. Thus, we chose 31°C as the “cool” temperature to mimic the physiological conditions under which adipocytes reside *in vivo*. MSCs were cultured at 37°C prior to and for the first 4 days of adipogenesis, after which cells were moved to 31°C for 12 days. Exposure to 31°C does not influence the late stages of adipogenesis, as assessed by cell morphology and expression of adipocyte markers, such as PPARγ, adiponectin, and FABP4 (S2A and S2B Fig). Importantly, beige adipocyte markers, including *Ucp1*, *Fgf21*, and *Pgc1a*, are not induced in white adipocytes cultured at 31°C (S2B and S2C Fig). Consistent with our *in vivo* results (Fig 1), expression of SCD1 mRNA and protein is increased in adipocytes cultured at temperatures below 37°C (Fig 2A and 2B). Indeed, changes in SCD1 expression are observed with shifts as small as 2°C, from 37°C to 35°C and particularly from 35°C to 33°C. Effects of culturing cells at 31°C on SCD1 protein emerge as early as 12 hours after exposure, with further increases observed until 96 hours (Fig 2D); in contrast, effects on *Scd1* mRNA are more subtle, only becoming significant after exposure to 31°C for 96 hours (Fig 2C), suggesting that the primary mechanism of regulation is protein synthesis or stability. As expected, adipocytes cultured at 31°C for 11 days have increased unsaturation of TAG lipids, characterized by a significant switch from palmitic to palmitoleic acid (Fig 2E), with milder changes observed in composition of phospholipids. Similarly, a higher proportion of double bonds are observed in TAG following cool adaptation (Fig 2F). Taken together, these data indicate that effects of cool temperature on induction of SCD1 and increased lipid monounsaturation are cell autonomous.

### Exposure of adipocytes to 31°C activates an extensive transcriptomic program of adaptation

**Fig 3:**
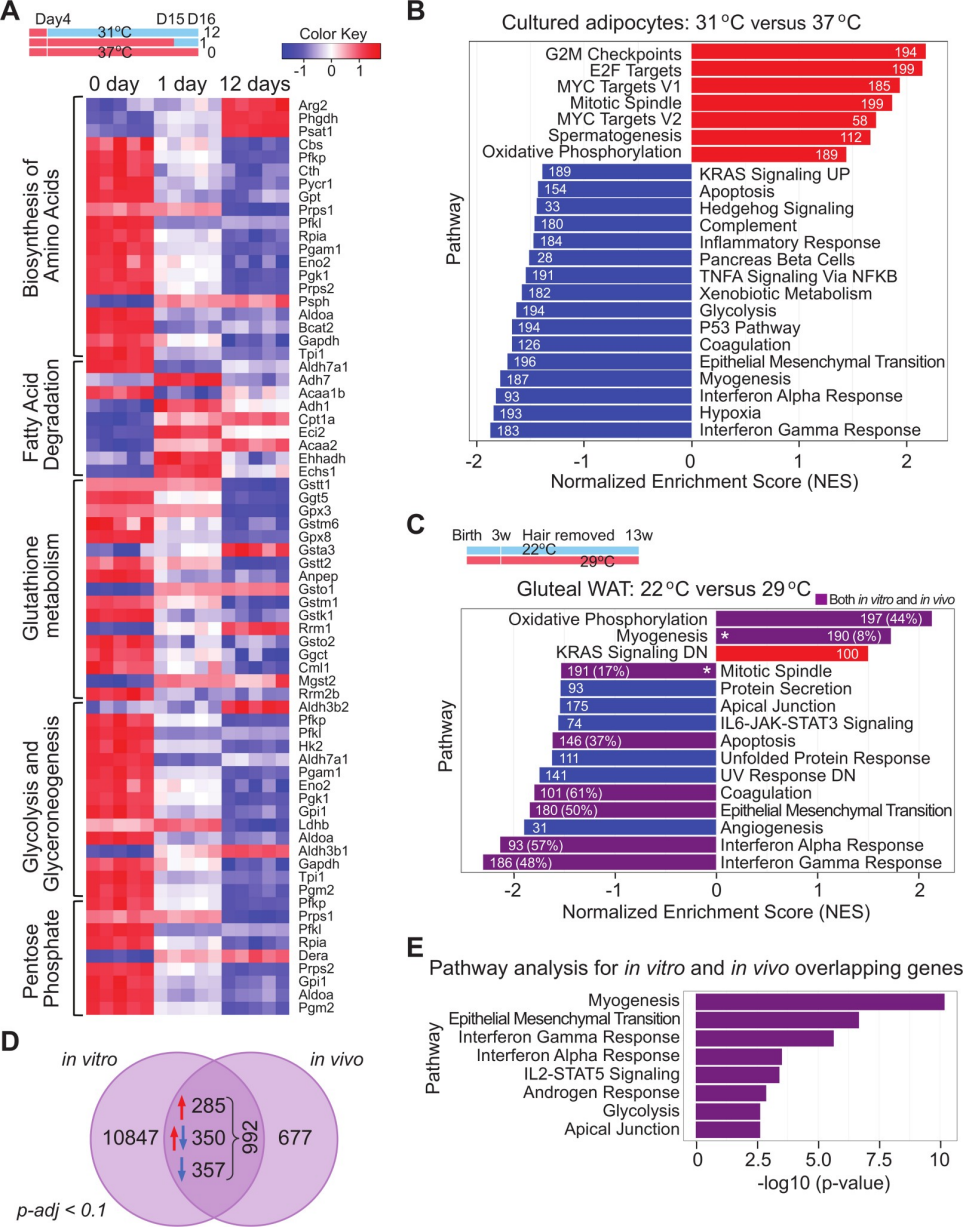
Exposure of adipocytes to 31°C activates an extensive transcriptomic program of adaptation. (**A, B**) RNA from mature adipocytes at day 0, 1, and 12 of cool adaptation was purified and subjected to RNA-seq analyses (*n =* 5 per time point) (**A**). Heat map of select metabolic KEGG pathways significantly enriched by 1 or 12 days of cool temperature from iPathwayGuide pathway impact analysis. Enrichment was defined by a nominal *p*-value < 0.05 and FDR < 0.25. (**B, C**) Normalized score plots of enriched pathways from preranked GSEA comparing RNA expression in mature adipocytes exposed to 31°C vs. 37°C for 12 days (**B**), or in gluteal WAT of mice housed at 22°C vs. 29°C for 13 weeks, as described in Fig 1C (*n* = 6, 7) (**C)**. Enriched pathways were defined by a nominal *p*-value < 0.05 and FDR < 0.05. *p*-Values were calculated from GSEA with 1,000 permutations. Numbers in the bars indicate gene set size. Purple bars indicate gene sets shared between datasets, and % indicates proportion of shared leading edge genes with the *in vitro* data. * indicate common pathways between the *in vitro* and *in vivo* data that are regulated in opposing directions. Data shown are from one experiment. (**D**) Venn diagram indicating the numbers of genes similarly regulated (P-adj < 0.1) in cultured adipocytes and gluteal WAT by cool temperatures. (**E**) Pathways identified by Enrichr software from genes regulated in both datasets. Numerical data for all graphs are provided in S3 Data. FDR, false discovery rate; GSEA, gene set enrichment analysis; RNA-seq, RNA sequencing; WAT, white adipose tissue.

To evaluate the transcriptomic program underlying cool adaptation, we performed RNA sequencing (RNA-seq) on white adipocytes exposed to 31°C for 0, 1, or 12 days. Twelve days of cool adaptation causes large-scale changes in gene expression, with up-regulation of 1,872 genes and suppression of 2,511 genes (S3 Fig). However, expression of common adipocyte genes such as *Cebpa*, *Creb*, *Klfs*, *Glut4*, *Plin*, *Lpl*, and *Bscl* are not altered by either 1 or 12 days of cool exposure (NCBI GEO: GSE159451 https://www.ncbi.nlm.nih.gov/geo/query/acc.cgi?acc=GSE159451), consistent with lack of obvious morphological differences. Markers of brown and beige adipocytes (e.g., *Ucp1*, *Ppargc1a*, *Eva1*, *Tnfsf9*, *Cox8b*, and *Fgf21*) [19] are also not induced by cool exposure in these cells. Similarly, genes involved in the creatine kinase cycle (*Slc6a8*, *Gamt*, *Ckmt1*, and *Ckmt2*) [8] or the SERCA2b-dependent futile cycle (*ATP2a2*) [10] are not altered by exposure to 31°C for 1 or 12 days (NCBI GEO: GSE159451). Further, evaluation of genes involved in metabolism suggests that cool exposure results in altered amino acid and glutathione metabolism, suppressed glycolysis/gluconeogenesis and pentose shunt pathways, and elevated lipid degradation (Fig 3A). Gene set enrichment analysis (GSEA) revealed that 12 days of 31°C exposure significantly induces genes involved in oxidative metabolism, as well as cell cycle regulation, including E2F and Myc targets, mitotic spindle, and G2M checkpoints. GSEA also predicts that cool adaptation suppresses glycolysis, along with impaired response to interferon gamma and hypoxia (Fig 3B). Thus, exposure of primary cultured adipocytes to 31°C for 1 or 12 days results in extensive changes to the transcriptome, suggesting that non-glucose energy sources support elevated oxygen consumption.

To determine whether the transcriptomic program of cool adaptation observed in cultured adipocytes is shared with adipose tissues, we housed mice at 22°C or 29°C from birth to 13 weeks of age. We performed RNA-seq on samples isolated from the gluteal adipose depot (NCBI GEO: GSE169142 https://www.ncbi.nlm.nih.gov/geo/query/acc.cgi?acc=GSE169142) because this tissue has little susceptibility to form beige adipocytes under these environmental conditions and thus is ideal for modeling WAT found in humans. GSEA revealed that 8 of the gene sets observed in cultured cells, including oxidative phosphorylation (OXPHOS), are also regulated in gluteal WAT (Fig 3C). Comparison of genes regulated by cool temperatures in cultured adipocytes and gluteal adipose tissue reveal that approximately 65% (642) of genes are induced or repressed similarly, whereas approximately 35% (350) of genes are regulated in opposing directions (Fig 3D). Pathway analyses with Enrichr software on the set of genes regulated in both datasets by cool temperatures independently identified a number of pathways detected by GSEA, including epithelial mesenchymal transition and glycolysis (Fig 3E). Coordinate regulation of mRNAs and pathways in cultured adipocytes and adipose tissue provides further evidence for a cell autonomous program of adaptation to cool environmental temperatures.

### Adipocytes adapted to 31°C have increased oxygen consumption rates (OCRs) and altered nutrient selection and utilization

**Fig 4:**
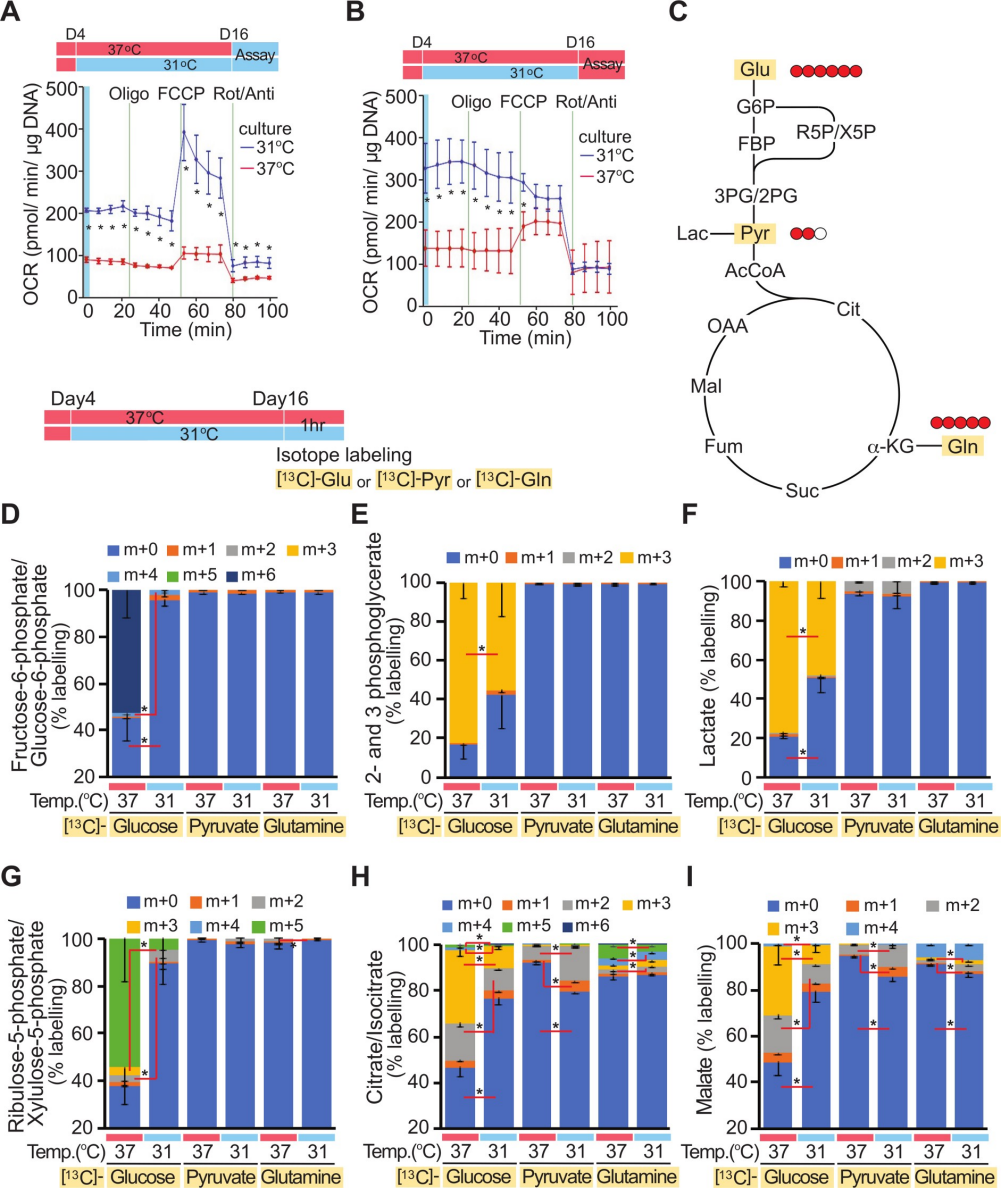
Adipocytes adapted to cooler temperatures have increased OCR and preferentially consume pyruvate and glutamine over glucose. (**A, B**) Seahorse XF96 Cell Culture Microplates were cut in half. Four days after adipocyte differentiation at 37°C, adipocytes on one half of the plate were cultured at 31°C and the other half at 37°C for 12 days. After rejoining the plate, OCR was evaluated either at 31°C (**A**) or 37°C (**B**) (*n* = 4). Data shown are representative of at least 3 independent experiments. (**C**–**I**) Differentiated adipocytes cultured at either 37°C or 31°C for 12 days were incubated with [^13^C]-glucose, [^13^C]-pyruvate, or [^13^C]-glutamine for 1 hour. Substrate concentrations in media were kept constant except for the substitution of [^13^C]-metabolites (5 mM glucose, 0.2 mM pyruvate, and 1 mM glutamine). Color code indicates [^13^C]-labeling on 0 through 6 carbons. Values are mean ± SD (*n* = 3). *p < 0.05. Data shown are from one experiment. Uncropped western blots are provided in S1 Raw Images, and numerical data for all graphs are provided in S4 Data. OCR, oxygen consumption rate; Oligo, oligomycin; Rot/Anti, rotenone and antimycin.

To test predictions from RNA-seq analyses, we next evaluated OCR of cool-adapted adipocytes using a Seahorse XF Analyzer (Fig 4A and 4B). To control for potential effects of short-term temperature changes on oxidative processes, cool-exposed and control adipocytes were analyzed at both 37°C and 31°C. Cool adaptation increases basal OCR approximately 2.5-fold when evaluated at either temperature (Fig 4A and 4B). Mitochondrial capacity for respiration is elevated when cool-adapted adipocytes are evaluated with FCCP at 31°C (Fig 4A); however, this difference is masked at 37°C by an increase to near-maximal rates of oxidative metabolism (Fig 4B). Of note, adipocytes cultured at 37°C also exhibit reduced rates of metabolism when assayed at 31°C rather than at 37°C. In cool-adapted adipocytes assayed at 31°C, we observed elevated non-mitochondrial OCR following treatment with rotenone/antimycin, which is generally attributed to other cellular oxidative reactions, including peroxisomal respiration; however, this difference is not observed in cells assayed at 37°C.

We next evaluated which substrates are utilized for increased oxygen consumption in adipocytes adapted to 31°C. RNA-seq and pathway analyses suggested that flux through glycolysis and the pentose phosphate shunt would be suppressed with cool adaptation (Fig 3); thus, we performed metabolic flux analyses using isotope-labeled glucose, pyruvate, and glutamine as carbon tracers. Adipocytes were cultured at 31°C or 37°C for 12 days and then incubated for 1 hour with [^13^C]-glucose, [^13^C]-pyruvate, or [^13^C]-glutamine (Fig 4C). Cool-adapted adipocytes have reduced flux of glucose through glycolysis, as assessed by [^13^C]-glucose labeling of fructose-6-phosphate/glucose-6-phosphate (Fig 4D), 2- and 3-phosphoglycerate (Fig 4E), and lactate (Fig 4F), and through the pentose phosphate shunt, as assessed by [^13^C]-glucose labeling of ribulose-5-phosphate and xylulose-5-phosphate (Fig 4G). There is also reduced flow of [^13^C]-glucose into tricarboxylic acid cycle intermediates, such as citrate/isocitrate (Fig 4H) and malate (Fig 4I). In contrast, a significant proportion of labeled carbons from [^13^C]-pyruvate and [^13^C]-glutamine are incorporated into citrate/isocitrate and malate (Fig 4H and 4I). Thus, adaptation to 31°C increases basal OCR, and both transcriptomic and metabolomic flux analyses point to diminished reliance on glucose for oxidative metabolism. Further, these results suggest that adipocytes adapted to cooler temperatures preferentially consume pyruvate and glutamine over glucose.

### Fatty acid oxidation is increased in cultured adipocytes adapted to cool environmental temperatures

**Fig 5:**
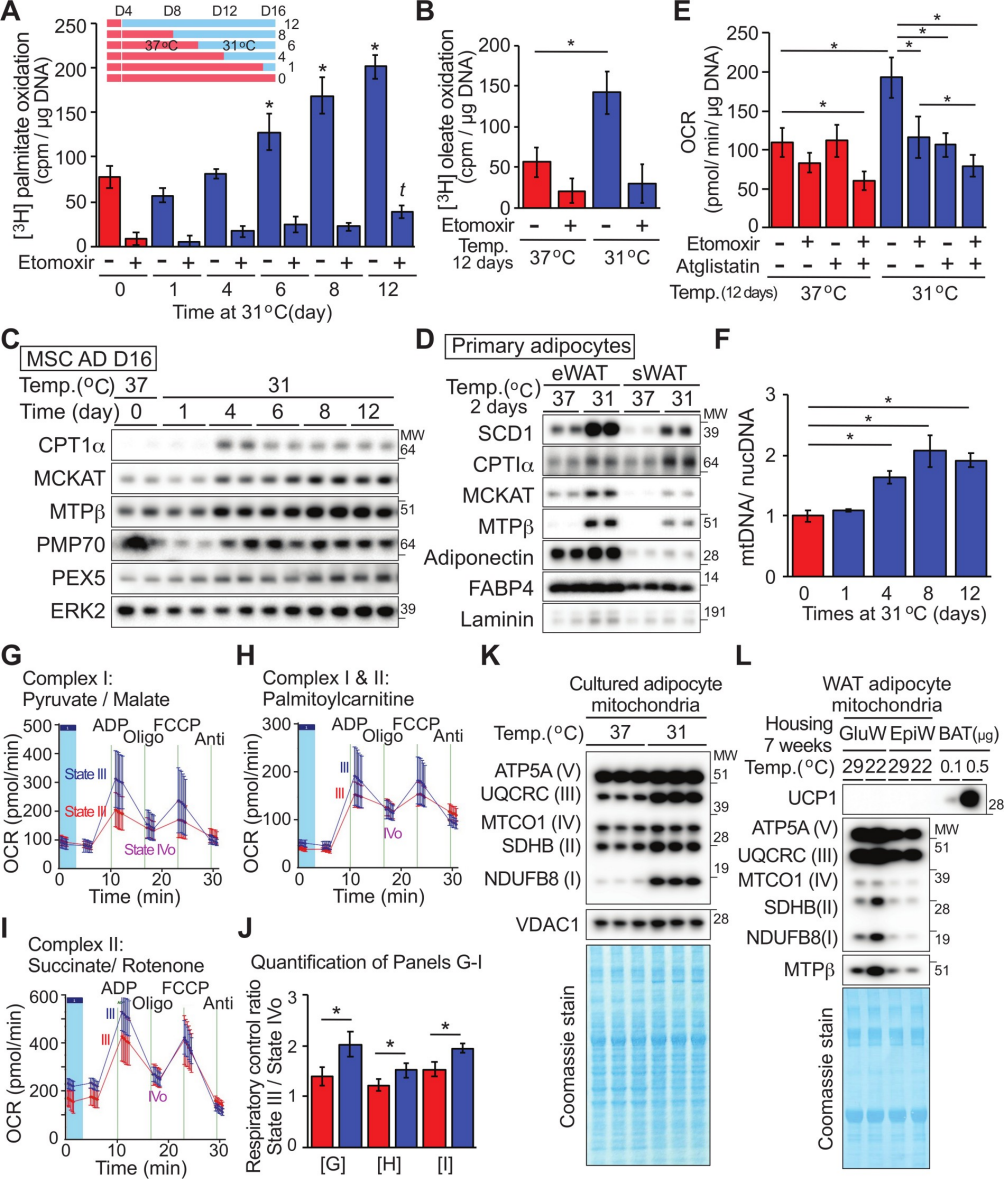
Cool adaptation of adipocytes stimulates β-oxidation of fatty acids and increases number and functioning of mitochondria. (**A, B**) Adipocytes at indicated days of cool temperature exposure (**A**; *n* = 5) or for 12 days (**B**; *n* = 6) were incubated with tritiated palmitate (**A**) or oleate (**B**) for 3 hours +/− 40 μM etomoxir. * significantly different from day 0 control at 37°C; *p* < 0.05). *t* indicates a difference from day 0 etomoxir treatment. (**C**) Cool adaptation increases mitochondrial fatty acid oxidation and peroxisomal proteins. (**D**) Primary adipocytes isolated from eWAT or sWAT were cultured in suspension at either 37°C or 31°C for 2 days. Expression of SCD1, CPT1α, MCKAT, and MTPβ was then determined by immunoblot analyses; adiponectin, FABP4, and laminin were loading controls. (**E**) Elevated basal OCR of adipocytes at 31°C is partially dependent on oxidation of endogenous fatty acids. MSC-derived adipocytes cultured at 31°C or 37°C for 12 days were treated with vehicle, 20 μM etomoxir and/or 20 μM of atglistatin for 2 hours prior and during assay of OCR (*n* = 8–10). (**F**) Four-day exposure at 31°C stimulates an increase in mitochondrial number as assessed by qPCR for 2 mitochondrial regions (CytoB, Cox-TMS) and 2 nuclear genes (glucagon, β-globin; *n* = 3). (**G**–**I**) Isolated mitochondria from adipocytes adapted to 31°C have improved functioning of complex I and II. Mitochondria were incubated with media containing pyruvate/malate for NADH-linked (complex I) substrates (**G**), Palmitoylcarnitine/malate for complexes I and II (**H**), and succinate/rotenone for complex II (**I**). (**J**) Calculated RCRs of mitochondria from panel (**G**) to (**I**), (*n* = 3 per group), values are mean ± SD. *p < 0.05. (**K**) Isolated mitochondria were lysed, and proteins were quantified. OXPHOS proteins were evaluated by immunoblot. VDAC1 is used as a loading control and Coomassie blue staining of the membrane shows other mitochondrial proteins. (**L**) Mitochondria were isolated from floating adipocytes following collagenase digestion of gluteal or eWAT from 5 mice housed at either at 29°C or 22°C for 7 weeks. OXPHOS proteins were evaluated and Coomassie staining was performed. Data are presented as mean ± SD. *p < 0.05. Data shown are representative of at least 3 independent experiments. Uncropped western blots are provided in S1 Raw Images, and numerical data for all graphs are provided in S5 Data. CPT1α, carnitine palmitoyltransferase 1α; CytoB, cytochalasin B; eWAT, epididymal white adipose tissue; FABP4, fatty acid-binding protein 4; MCKAT, medium-chain 3-ketoacyl-CoA thiolase; MSC, mesenchymal stem cell; MTPβ, mitochondrial trifunctional protein beta subunit; OCR, oxygen consumption rate; OXPHOS, oxidative phosphorylation; qPCR, quantitative polymerase chain reaction; RCR, respiratory control ratio; SCD1, stearoyl-CoA desaturase-1; sWAT, subcutaneous white adipose tissue; VDAC1, voltage-dependent anion-selective channel 1; WAT, white adipose tissue.

Adipocytes adapted to 31°C shift carbon sources away from glucose toward glutamine and pyruvate (Fig 4). Although amino acids are anaplerotic substrates in adipocytes [20], glucose and fatty acids are considered to be primary carbon sources for their energy needs. To explore further how cool adaptation influences adipocyte lipid metabolism, we measured β-oxidation in adipocytes exposed to 31°C for different lengths of time. We observed that release of tritiated water from adipocytes treated with [^3^H]-palmitic acid is elevated after 6 days of exposure to 31°C and increases further with 8 and 12 days of cool adaptation (Fig 5A). Increased β-oxidation in cool-adapted adipocytes is also observed when labeled oleic acid is used as a substrate (Fig 5B). Interestingly, oxidation of octanoic acid (C8:0; S4A Fig) is only slightly increased with cool adaptation, suggesting that mitochondrial uptake of fatty acids is highly regulated by cool adaptation. In this regard, proteins involved in both uptake and β-oxidation of fatty acids, including carnitine palmitoyltransferase 1α (CPT1α), medium-chain 3-ketoacyl-CoA thiolase (MCKAT), and mitochondrial trifunctional protein beta subunit (MTPβ), are up-regulated by cool adaptation; these effects are observed in several cell models, including cultured adipocytes differentiated from MSCs (Fig 5C) or stromal vascular cells (SVCs; S4B and S4C Fig), and in primary white adipocytes isolated by collagenase digestion of WAT (Fig 5D). Pharmacological inhibition of mitochondrial fatty acid uptake (Fig 5A) or OCR (Fig 4A and 4B) indicates that there is also a significant non-mitochondrial component involved in cool adaptation. This may be due to elevated peroxisomal α-, or β-oxidation, as evidenced by increased expression of peroxisome markers, such as PMP70 and PEX5 (Fig 5C). Since peroxisomal oxidation of fatty acids is not coupled to ATP synthesis, it may contribute to heat generation in cool-adapted adipocytes.

These results led us to investigate further the relationship between fatty acid utilization and increased OCR in adipocytes cultured at 31°C. Pharmacologic inhibition of endogenous fatty acid uptake into mitochondria with the CPT1 inhibitor, etomoxir, decreases basal OCR of cool-adapted adipocytes by approximately 50%, whereas the OCR of adipocytes cultured at 37°C is not affected (Fig 5E). Since the assay media does not contain exogenous fatty acids, treatment with etomoxir exclusively blocks entry of endogenous fatty acids into the mitochondria. Similarly, treatment of cool-adapted adipocytes with the adipose triglyceride lipase (ATGL)-specific inhibitor aglistatin blocks lipolysis, thereby decreasing the endogenous fatty acid supply and suppressing basal OCR of cool-adapted adipocytes (Fig 5E). Although combined treatment with etomoxir and atglistatin suppresses basal OCR in cells cultured at either 31°C or 37°C, we observed a greater degree of suppression in cool-adapted adipocytes, suggesting that these cells rely more heavily on endogenous fatty acids for energy.

### Isolated mitochondria from adipocytes adapted to 31°C have improved complexes I and II function

Increased OCR and fatty acid oxidation in cool-adapted adipocytes led us to next investigate effects on mitochondrial number and function within these cells. Measurement of mitochondrial DNA (mtDNA) relative to nuclear DNA (ncDNA) by qPCR demonstrated that mitochondrial number doubles by 8 days of cool adaptation (Fig 5F). Furthermore, mRNA expression of most mitochondrial complex subunits is also up-regulated following exposure to 31°C (S4D Fig). This mitochondrial expansion is not accompanied by increased expression of *Ppargc1a*, *Tfam*, *Nrf1*, or *Ucp1* (NCBI GEO: GSE159451), although up-regulation of mitochondrial proteins specifically involved in the β-oxidation pathway is observed (Fig 5C and 5D). Although elevated capacity for cellular OCR and fatty acid oxidation with adaptation to 31°C can be explained, in part, by an increase in mitochondrial number (Fig 5F), we also evaluated functional characteristics of isolated mitochondria. Mitochondrial respiratory control encapsulates the main function of mitochondria: the ability to idle at a low rate and respond to ADP to generate large quantities of ATP. We found that ADP-stimulated respiration (State III) is higher in mitochondria from adipocytes cultured at 31°C compared to those cultured at 37°C (Fig 5G–5J). These results indicate that mitochondria of cool-adapted adipocytes have enhanced function of both complex I (malate plus pyruvate) (Fig 5G and 5J) and complex II (succinate plus rotenone) (Fig 5I and 5J). These results also hold true when mitochondria are treated with palmitoylcarnitine to test complexes I and II together (Fig 5H and 5J). No significant changes in non-ADP-stimulated respiration (State IVo) were observed between the 2 groups following inhibition of ATP synthase by oligomycin. These data suggest that cool-adapted adipocyte mitochondria have a high capacity for substrate oxidation and ATP turnover. Immunoblot analyses of OXPHOS complexes revealed that NDUFB8 of complex I, SDHB of complex II, and UQCRC2 of complex III are increased disproportionately when compared to other mitochondrial proteins evaluated (Fig 5K).

Next, to examine whether expression of specific OXPHOS proteins is regulated by temperature *in vivo*, mice were housed at 29°C or 22°C for 7 weeks. Mitochondria were then isolated from floating adipocytes following collagenase digestion of subcutaneous gluteal WAT or visceral epididymal white adipose tissue (eWAT). Immunoblot analyses revealed that expression of complex I (NDUFB8) and complex II (SDHB) proteins are elevated in mitochondria isolated from gluteal WAT of mice housed at 22°C (Fig 5L), whereas no differences are observed in other complex proteins. These results suggest that in cool-adapted adipocytes, increased oxidative metabolism is due not only to increased mitochondrial number but also to formation of more high-functioning, “healthy” mitochondria with improved respiratory capacity. Of note, elevated uncoupled mitochondrial respiration in cool-adapted adipocytes is not due to canonical adaptive thermogenesis, since *Ucp1* mRNA expression is unchanged (NCBI GEO: GSE159451) and protein levels are undetectable in both cultured and primary white adipocytes (S5A and S5B Fig). Furthermore, increased OCR in response to cool exposure occurs at similar levels in wild-type and UCP1-deficient adipocytes (S5D Fig), with comparable induction of proteins such as SCD1, FASN, and CPT1α (S5C Fig).

### Adaptation to 31°C for 12 days down-regulates basal and stimulated TAG hydrolysis but does not limit total lipolytic capacity

**Fig 6:**
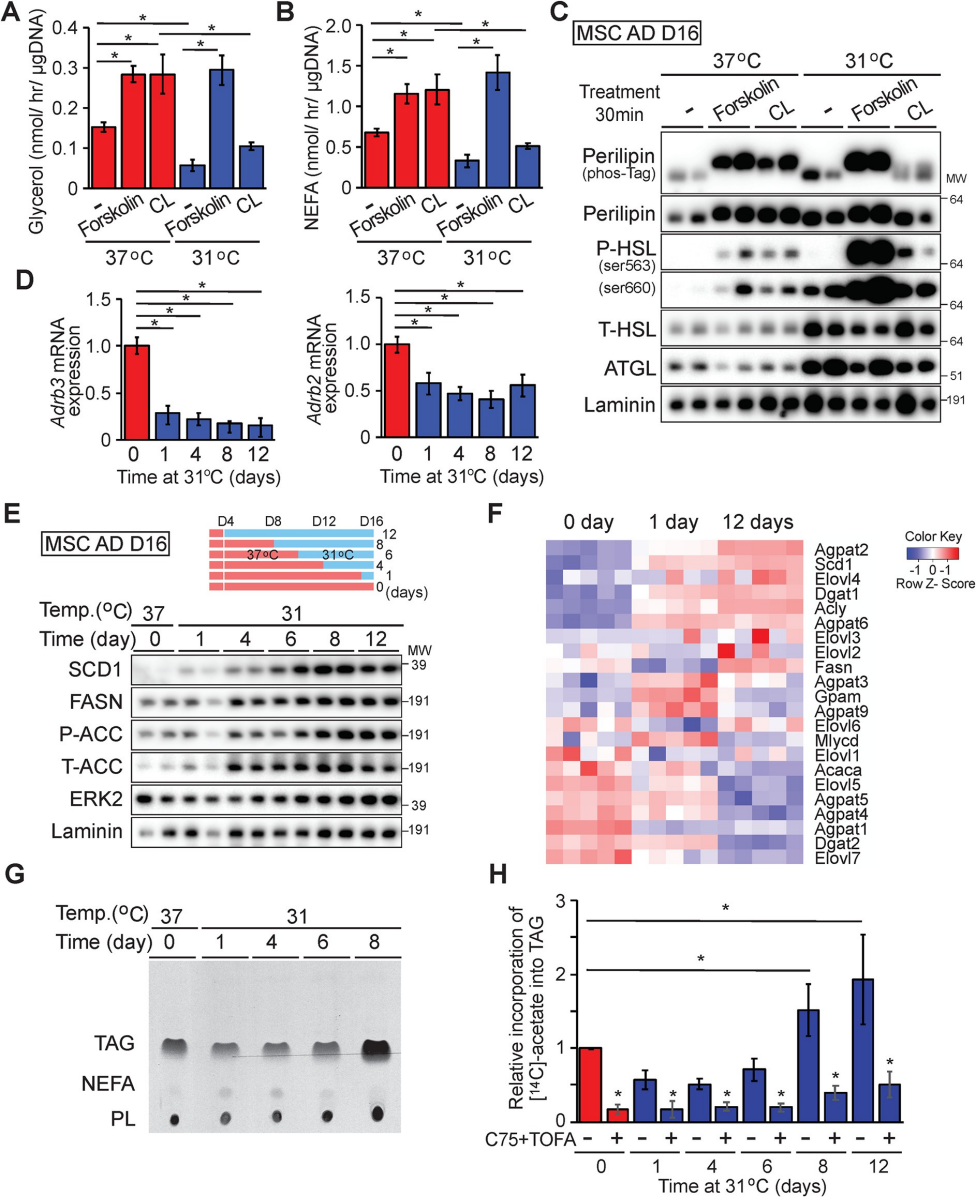
Adaptation to cool temperature decreases basal and stimulated lipolysis and increases *de novo* lipogenesis and TAG synthesis. (**A, B**) Adipocytes were treated with 10 μM forskolin or 2 μM CL-316,243 (CL) for 6 hours and secretion of glycerol (**A**) and NEFA (**B**) measured (*n* = 4). (**C**) Cool adaptation increases expression of lipases but decreases phosphorylation of perilipin and HSL in response to CL-316,243. Adipocytes were treated with vehicle, 10 μM forskolin, or 2 μM CL for 30 minutes and lysed for immunoblot analyses. (**D**). Rapid reduction of β3- and β2-adrenergic receptor mRNAs after exposure to 31°C (*n* = 6 per time point). Gene expression was normalized to geometric mean of *Hprt*, *Tbp*, and *Ppia* and is expressed relative to 37°C control (*n* = 4). (**E**) Expression of *de novo* lipogenesis-related proteins in adipocytes cultured at 31°C. (**F**) Relative expression of *de novo* lipogenesis-related mRNAs after 0, 1, or 12 days of 31°C. Genes were manually curated since *de novo* lipogenesis is not captured specifically by iPathwayGuide or GSEA. Cool adaptation for 8 days increases incorporation of [^14^C]-acetate into TAG and phospholipids. (**G**) [^14^C]-acetate was added to media of adipocytes cultured at 31°C. Metabolites were separated by thin layer chromatography, and [^14^C] was detected by autoradiography. Addition of 10 μM TOFA and 50 μM of C75 was for 3 hours before and throughout the lipogenesis assay. (**H**) Quantification of autoradiography for labeled TAG was by ImageJ. *n =* 5, data are mean ± SD. *p < 0.05. Uncropped western blots are provided in S1 Raw Images, and numerical data for all graphs are provided in S6 Data. ACC, acetyl-CoA carboxylase; ATGL, adipose triglyceride lipase; GSEA, gene set enrichment analysis; FASN, fatty acid synthase; HSL, hormone-sensitive lipase; NEFA, nonesterified fatty acid; PL, phospholipid; TAG, triacylglycerol; TOFA, 5-(tetradecyloxy)-2-furoic acid.

RNA-seq analyses identified *Adrb3* as one of the most profoundly suppressed adipocyte genes with cool exposure (S3A Fig). *Adrb3* encodes the β_3_-adrenergic receptor (β_3_-AR), which is predominantly expressed in rodent adipocytes [21]. Activation of β-AR leads to the activation of protein kinase A, which phosphorylates perilipin and HSL to promote lipolysis. Thus, we next investigated effects of cool temperatures on basal and stimulated lipolysis. Adipocytes exposed to 31°C or 37°C for 12 days were treated with vehicle, forskolin, or the β_3_-agonist CL-316,243 (CL) for 6 hours. Lipolysis was evaluated by measurement of nonesterified fatty acids (NEFAs) and glycerol released into media. As expected, adipocytes cultured at 37°C respond robustly to both forskolin and CL by increasing release of glycerol and NEFA (Fig 6A and 6B). In contrast, cool-adapted adipocytes demonstrate significantly reduced lipolysis at baseline and following stimulation with CL. Adipocytes cultured at 31°C and 37°C respond similarly to forskolin treatment, suggesting that reduced secretion of lipolytic products from cool-exposed adipocytes at baseline or in response to CL is not due to decreased lipolytic capacity (Fig 6A and 6B).

To explore the molecular bases for altered lipolytic response of cool-adapted adipocytes, we performed immunoblot analyses and observed elevated expression of ATGL and HSL in adipocytes cultured at 31°C (Fig 6C). Increased expression of lipolytic enzymes may seem paradoxical, given the observed reduction in basal lipolysis in cool-adapted cells; however, a lack of correlation between expression of ATGL or HSL and basal lipolysis has been observed previously [22]. As expected, treatment of adipocytes cultured at 37°C with forskolin or CL increases phosphorylation of HSL at Ser563 and Ser660 (Fig 6C, S6A and S6B Fig). In cool-adapted adipocytes, we observed an excellent overall correlation between HSL phosphorylation and lipolysis—the increased phosphorylation of HSL at baseline is proportional to total HSL, and phosphorylation is substantially increased following treatment with forskolin but not CL.

Regulation of lipolysis is also dependent upon phosphorylation of perilipin by protein kinase A, which releases ABHD5 to coactivate ATGL [23]. In this regard, analyses using a Phos-Tag gel system reveal that perilipin is dephosphorylated at baseline in adipocytes cultured at either temperature. However, whereas perilipin phosphorylation is increased by both forskolin and CL treatment in adipocytes cultured at 37°C, elevated phosphorylation is only observed with forskolin in adipocytes exposed to 31°C (Fig 6C, S6A and S6B Fig). One reason for impaired responsivity of cool-adapted adipocytes to CL is reduced expression of β_3_-AR, which is rapidly suppressed by exposure to 31°C (S3A Fig, Fig 6D). In addition, β_2_-adrenergic receptor expression is also rapidly suppressed (Fig 6D), suggesting a general resistance to adrenergic stimuli in cool-adapted cells. Taken together, exposure of adipocytes to 31°C blunts β3-AR expression and β_3_-AR-dependent lipolysis, but adaptation does not limit total lipolytic capacity.

### Adaptation to cool temperatures increases *de novo* lipogenesis and TAG synthesis

We next explored effects of cool adaptation on anabolic aspects of lipid metabolism. In addition to SCD1, exposure to 31°C up-regulates expression of other lipogenic proteins, such as fatty acid synthase (FASN) and acetyl-CoA carboxylase (ACC) (Fig 6E). Of note, *Fasn* mRNA expression is initially decreased after 1 day at 31°C but is subsequently increased at 12 days of exposure (Figs 3 and 6F). To evaluate functional effects of increased lipogenic gene expression with cool exposure, we incubated adipocytes cultured at 37°C or 31°C with [^14^C]-acetate for 12 hours (Fig 6G and 6H). Rates of *de novo* lipogenesis increased over 8 days of cool adaptation, with increased radiolabel incorporation into TAG fractions of cellular lipid. Consistent with increased TAG synthesis in adipocytes adapted to 31°C, expression of *Dgat1*, which catalyzes the terminal step of TAG synthesis, is also elevated (Fig 6F and NCBI GEO: GSE159451). Taken together, these results indicate that cool adaptation up-regulates lipogenic gene and protein expression and functionally increases *de novo* lipogenesis capacity and rates of TAG synthesis. Many *de novo* lipogenesis genes are regulated transcriptionally in adipocytes by sterol regulatory element-binding protein-1c (SREBP-1c) and carbohydrate response element-binding protein (ChREBP) [24]. Using loss-of-function approaches, we found that increased proteins involved in *de novo* lipogenesis in response to cool adaptation are regulated independently of these well-known transcription factors (S6C and S6D Fig).

### SCD1 activity is required for adipocytes to metabolically adapt to 31°C

**Fig 7:**
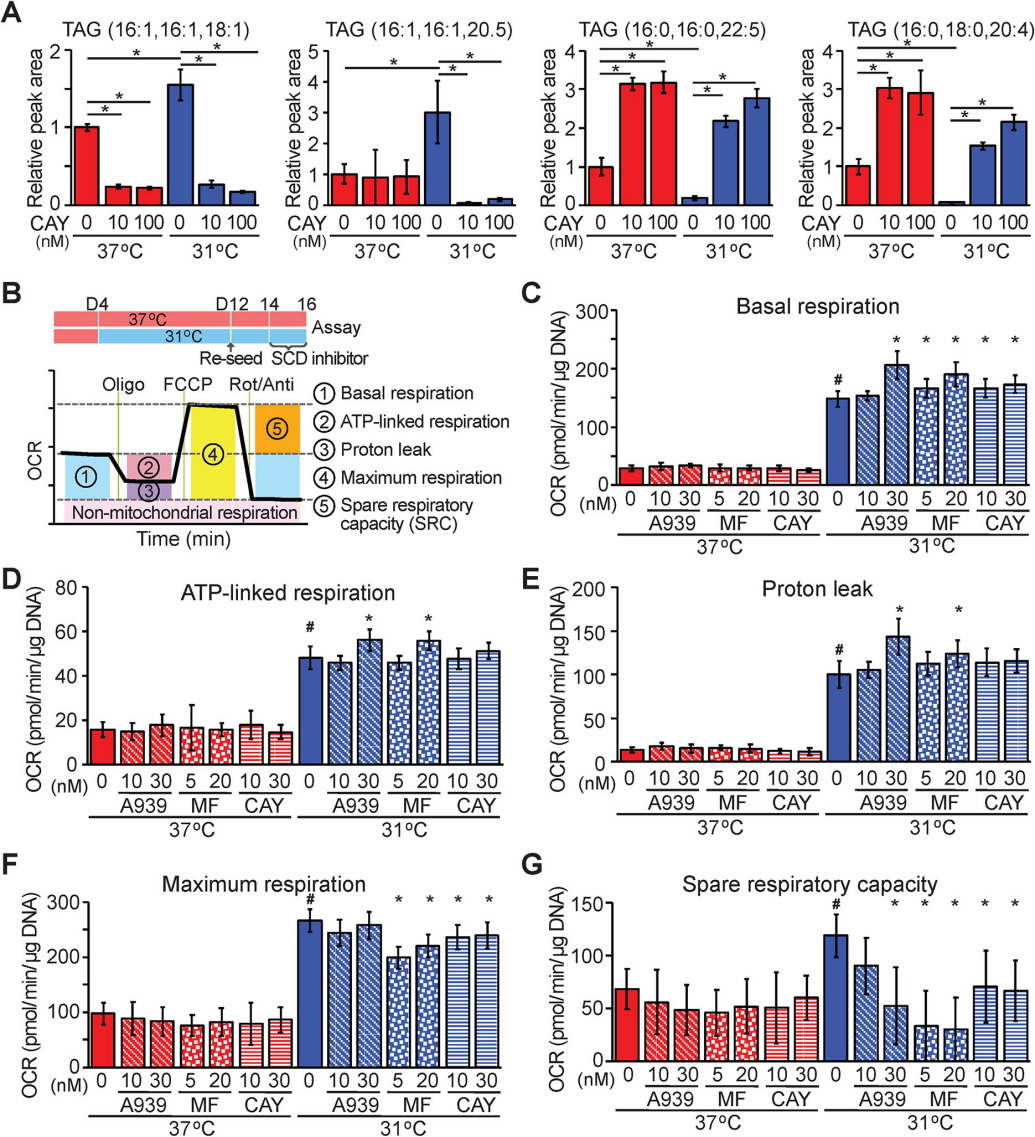
SCD1 activity is required for adipocytes to increase maximal OCR and spare respiratory capacity during adaptation to cool temperature. (**A**) CAY10566 decreases TAG containing monounsaturated lipids (left panels) and increases TAG containing saturated lipids (right panels). Adipocytes were cultured at 37°C or 31°C for 10 days with indicated concentrations of CAY10566. Peak area of each lipid is expressed as fold change relative to 37°C control (*n* = 4). Data are presented as mean ± SD. *p < 0.05. (**B**–**G**) Differentiated adipocytes cultured at either 37°C or 31°C for 8 days were trypsinized, and adipocytes were isolated by centrifugation. Adipocytes were seeded on Cell-Tak coated Seahorse XF96 microplates, then cultured overnight upside down at either 37°C or 31°C. Adipocytes were cultured with SCD inhibitors: A-939572, MF-438, or CAY10566 at the indicated temperature for 2 days before assay (*n* = 8–16 per group). (**B**) Summary of metabolic variables estimated. (**C**) Basal respiration. (**D**) ATP-linked respiration. (**E**) Proton leak. (**F**) Maximal respiration. (**G**) Spare respiratory capacity. Values are mean ± SD. * is significantly different from vehicle control. *#* indicates a difference from 37°C vehicle control. Data shown are representative of at least 3 independent experiments. Numerical data for all graphs are provided in S7 Data. FCCP, carbonyl cyanide-*p*-trifluoromethoxyphenylhydrazone; OCR, oxygen consumption rate; Oligo, oligomycin; Rot/Anti, rotenone and antimycin; SCD1, stearoyl-CoA desaturase-1; SRC, spare respiratory capacity; TAG, triacylglycerol.

Our studies have revealed that SCD1 mRNA and protein levels are up-regulated in cool-adapted adipocytes both in culture and *in vivo* (Figs 1, 2 and 6). Further, we have shown that palmitic acid (C16:0), a substrate for SCD1, and oleic acid (C18:1), a product of this enzyme, are both oxidized at elevated rates in cool-adapted adipocytes (Fig 5). Thus, we next investigated the specific functional role of SCD1 in cool adaptation of adipocytes. Since cultured SCD1 knockout cells are prone to beiging [25] and have impaired lipid accumulation during adipogenesis, we used a pharmacological approach for our studies. Cool exposure of cultured adipocytes increases proportion of TAG species containing monounsaturated lipids at the expense of those containing saturated lipids, as assessed by liquid chromatography–mass spectrometry (LC–MS) (Fig 7A). Inhibition of SCD1 with CAY10566 (CAY) shifts lipid composition of TAG from species containing 16:1 monounsaturated lipids (Fig 7A; left 2 panels) toward species containing 16:0 and 18:0 saturated lipids (Fig 7A; right 2 panels), and these effects are largely independent of temperature. For adipocytes cultured at 37°C, inhibition of SCD1 activity with CAY10566, A-939572 (A939), or MF-438 (MF) for 2 days prior to the assay (Fig 7B) had no effect on OCR during Seahorse analyses (Fig 7C–7G, S7A Fig). However, for adipocytes adapted to 31°C, SCD1 inhibition increased basal respiration, ATP-linked respiration, and proton leak, and reduced maximum respiration. Thus, spare respiratory capacity, which is defined as maximal OCR minus basal OCR in cool-adapted adipocytes, is specifically reduced in adipocytes cultured at 31°C (Fig 7C–7G, S7B Fig). These data demonstrate that increased SCD1 activity is required for adipocytes to metabolically adapt to cooler temperatures.

## Discussion

### Elevated oxygen consumption with cool adaptation is partially fueled by newly synthesized and stored fatty acids

Adipocytes exposed to 31°C are characterized by elevated oxygen consumption, with increased anabolic and catabolic lipid metabolic processes working in parallel. Although flux of glucose through glycolysis and the pentose phosphate shunt is suppressed, use of other nutrients to fuel oxidative metabolism, including pyruvate, glutamine, and fatty acids, is increased. Interestingly, cool adaptation of white adipocytes up-regulates *de novo* lipogenesis and expression of SCD1; thus, newly synthesized fatty acids are more highly desaturated prior to incorporation into TAG. Based on inhibition with etomoxir and atglistatin, NEFA from endogenous stores are the primary energy source for elevated oxygen consumption in adipocytes at 31°C. Cool-adapted adipocytes have higher demands for fatty acids hydrolyzed from TAG; however, they are also characterized by reduced secretion of glycerol and NEFA. Thus, cool adaptation of adipocytes is characterized by increased *de novo* lipogenesis, perhaps with pyruvate and amino acids as a source of carbon. TAG is then subject to partial hydrolysis in cool-adapted adipocytes, and whereas the newly hydrolyzed fatty acids are shuttled to mitochondria for oxidation, the acylglycerols are reesterified to form TAG.

### Elevated SCD1 expression and monounsaturation of TAG lipids are cell autonomous and required for elevated oxidative metabolism with cool adaptation

Previous studies in bacteria, plants, and poikilothermic animals have reported that cool temperatures increase lipid desaturation, whereas warm temperatures increase proportions of saturated lipids [26–28]. This observation is largely considered to be due to homeoviscous adaptation, in which regulation of plasma membrane phospholipid desaturation is required to maintain appropriate viscosity and membrane function at various temperatures [29]. However, our study reveals that compared to rats raised at thermoneutrality (29°C), distal BMAT of rats housed at 22°C exhibits increases TAG lipid unsaturation, whereas phospholipid composition is unaltered. A comparable observation is found in subcutaneous WAT of shaved mice. In addition, a disproportionate increase in lipid monounsaturation in TAG of cultured adipocytes is observed at 31°C, suggesting that elevated SCD1 preferentially influences composition of fatty acids incorporated into TAG versus phospholipids. Importantly, we demonstrate that SCD1 activity is required for maximal respiration and spare reserve capacity of adipocytes adapted to 31°C. These findings extend historical literature, which revealed a temperature gradient across the backfat of sows, and revealed that soft lard from the outer, cooler layer of subcutaneous fat contains increased unsaturated lipids, particularly oleate [14]. Together, these findings suggest an evolutionarily conserved role for lipid desaturation of TAG in metabolic adaptation to cool cellular environments.

Oleic acid and palmitoleic acid have previously been reported to induce expression of OXPHOS subunits 3T3-L1 adipocytes and fatty acid oxidation in skeletal muscle cells [30,31]. One intriguing possibility is that the observed increases in OXPHOS subunits and β-oxidation are regulated by increased production of monounsaturated lipids with cool adaptation. Indeed, we observed a higher proportion of the SCD1 product oleic acid (C18:1, n-9) esterified in TAG of dTib and CV BMAT, which exist at temperatures lower than those found at the body core. In cultured adipocytes exposed to cool temperatures, it is the SCD1 product palmitoleic acid (C16:1, n-7) that is dramatically elevated. Coincident with improved mitochondrial function, we observed increased expression of OXPHOS subunits with cool adaptation. In our studies, complexes I, II, and III were up-regulated in adipocytes at 31°C, whereas previous work in differentiated 3T3-L1 cells reported that palmitoleic acid induced oxygen consumption and complexes II, III, and V [31].

A cool environmental temperature had more profound effects on lipid composition of TAGs than phospholipids in cultured adipocytes. Further, the increase in lipid unsaturation of phospholipids was detected *in vitro*, but changes were not observed *in vivo*, perhaps due to contributions from non-adipocytes within BMAT. Because it is difficult to isolate large numbers of BMAds from mice, we analyzed lipids isolated from whole BMAT, which contains a considerable number of hematopoietic, endothelial, and stromal cells. Whereas the majority of phospholipids detected by lipidomic analysis *in vitro* are from differentiated adipocytes that predominantly express SCD1, the phospholipid fraction in bone marrow has a substantial contribution from non-adipocytes, which dilute detection of changes to adipocytes phospholipid composition. This mechanism, along with intercellular or neural compensation, may also account for other differences between systems such as expression of ADRB3 and ADRB2, which were unchanged in the RNA-seq data *in vivo* but were strongly suppressed *in vitro*.

### Identification of adaptive thermal responses specific to white adipocytes

Previous studies using clonal, immortalized cell lines and bone marrow-derived MSCs have suggested that in adipocytes, cool temperatures can activate the expression of thermogenic genes, such as *Ucp1* and *Pgc1a*, in a cell autonomous manner [7,32]. In this work, we have demonstrated that white adipocytes isolated from the epididymal depot, and adipocytes differentiated from MSCs or SVCs from WAT, do not up-regulate expression of UCP1 in response to cool temperatures. Moreover, our RNA-seq dataset did not reveal induction of canonical browning/beiging genes in these cells. It is possible that the strategies used by specific adipocyte models to adapt to cool temperatures depend on the propensity of individual cell types for browning/beiging.

From an energy utilization viewpoint, cool-adapted adipocytes possess high fatty acid oxidation capacity and preferentially use fatty acids over glucose as a fuel source. This metabolic shift for white adipocytes is distinct from other types of adipocytes; brown/beige adipocytes are characterized by high glucose uptake and oxidative capacity in “glycolytic beige fat” [33], and both glucose and fatty acids are used as substrates in conventional thermogenic brown and beige adipocytes following cold stimulation [34–37]. Importantly, exposure to cool temperatures does not influence expression of genes that define the adipocyte phenotype, nor are there differences observed in adipocyte morphology, including the appearance of brown or beige characteristics. Thus, our results demonstrate that white adipocytes and WATs adapted to cool temperatures have unique molecular and metabolic characteristics that distinguish them from counterparts adapted to warmer temperatures and from brown/beige adipocytes. Our study suggests that cool environmental temperatures are an unappreciated factor in stimulating the distinct cellular and physiological features of adipocytes found within different WAT depots. These findings provide further evidence that environmental temperature is an important consideration in experimental design [38].

## Methods

### Animals

Sprague Dawley rats were obtained from Envigo (USA), and 16 females were bred. Two days after giving birth, 8 dams and their litters remained in thermal chambers at 22°C, and 8 were transferred to thermal chambers at 29°C (i.e., thermoneutrality). Eleven weeks later, male and female rats were killed. C57BL/6J mice (The Jackson Laboratory, Ellsworth, ME, USA) were bred in thermal chambers at 22°C or 29°C. Male mice were housed from birth to 13 weeks either at 22°C or at 29°C. After weaning, posterior mouse hair was removed using Nair. Mice were single housed with minimal bedding and no nesting materials. Subdermal temperature was measured of mice housed at 22°C or 29°C using a rodent thermometer BIO-TK8851 (Bioseb Lab Instruments, France) under anesthesia. Temperature measurement was finished within 5 minutes of anesthesia to avoid temperature-lowing effects. All animal studies were performed in compliance with policies of the University of Michigan Institutional Animal Care and Use Committee. The protocol number is PRO00009687.

### Cell culture

Primary MSCs were isolated from the ears of wild-type C57BL/6J, UCP1-KO (Ucp1^tm1Kz^/J) (The Jackson Laboratory), and ChREBP KO (kindly gifted by Dr. Lei Yin at the University of Michigan) mice as previously described [39]. At 2 days post-confluence, adipogenesis was induced with 0.5 mM methylisobutylxanthine, 1 μM dexamethasone, 5 μg/ml insulin, and 5 μM rosiglitazone (Cayman Chemical, Ann Arbor, MI, USA) in DMEM:F12 containing 10% FBS. From day 2 to 4 of differentiation, cells were fed with fresh DMEM:F12 medium containing 10% FBS, 5 μg/ml insulin, and 5 μM rosiglitazone. Thereafter, cells were maintained in DMEM:F12 containing 10% FBS.

### Reagents

Reagents used were as follows: adenosine 5′-diphosphate (Sigma-Aldrich, St. Louis, MO, USA), A-939572 (Cayman Chemical), CAY10566 (Cayman Chemical), Cell-Tak (Corning, Corning, NY, USA), CL-316,243 (Tocris, Minneapolis, MN, USA), C75 (Cayman Chemical), etomoxir (Cayman Chemical), forskolin (Tocris), MF-438 (Millipore Sigma, St. Louis, MO, USA), octanoic acid (Sigma-Aldrich), oleic acid (Sigma-Aldrich), rotenone (Tocris), palmitoleic acid (Sigma-Aldrich), palmitoyl carnitine (Sigma-Aldrich), pyruvate (Sigma-Aldrich), sodium palmitate (Sigma-Aldrich), and TOFA (Cayman Chemical). Additional information is available in S1 Table.

#### Adipocyte and stromal vascular cell fractionation

Gluteal WAT and eWAT were excised from mice, minced with scissors, and digested for 1 hour at 37°C in 2 mg/ml collagenase type I (Worthington Biochemical, Lakewood, NJ, USA) in Krebs-Ringer-HEPES (KRH; pH 7.4) buffer containing 3% fatty acid-free bovine serum albumin (BSA; Gold Biotechnology, St. Louis, New Jersey, USA), 1 g/L glucose, and 500 nM adenosine. The resulting cell suspensions were filtered through 100 μm cell strainers and centrifuged at 100 × *g* for 8 minutes to separate the stromal vascular fraction and the buoyant adipocytes. Floating adipocytes were washed and cultured with DMEM:F12 containing 10% FBS.

#### Isolation and differentiation of adipocyte precursors

Mesenchymal precursors were isolated from ears of mice of the indicated genotypes, as previously described [18]. Cells were maintained in 5% CO_2_ and DMEM/F12 1:1 media (Gibco; Invitrogen, Carlsbad, CA, USA) supplemented with 15% FBS (Atlas Biologicals, Fort Collins, CO, USA), primocin (InVivoGen, San Diego, CA, USA), and 10 ng/ml recombinant bFGF (PeproTech, Rocky Hill, NJ, USA). For induction of adipogenesis, recombinant bFGF was removed and replaced with 10% FBS containing 0.5 mM methylisobutylxanthine, 1 μM dexamethasone, 5 μg/ml insulin, and 5 μM troglitazone. On day 2, cells were fed 5 μg/ml insulin plus 5 μM troglitazone. On day 4 and every 2 days thereafter, cells were fed with 10% FBS. At day 4 of differentiation, plates of adipocytes were either kept at 37°C or moved to another incubator maintained at 31°C. Of note, since adipocyte culture media contains both sodium bicarbonate and HEFPES, pH changes are unlikely to contribute to temperature-induced effects on adipocyte gene expression.

#### Lipolysis

Cultured adipocytes were washed 1× with PBS and then incubated in Hanks’ balanced salt solution (HBSS; Thermo Fisher Scientific, USA) containing 2% BSA for 1 hour at 37°C. Cells were treated with forskolin or CL-316,243 for 6 hours. Secretion of glycerol and NEFA from cultured adipocytes into HBSS was determined with assay kits from Sigma-Aldrich (FG0100) and FUJIFILM Wako Diagnostics-NEFA Reagent (NEFA-HR [2]), respectively.

#### *De novo* lipogenesis assay

Cultured adipocytes were incubated overnight in fresh serum-free DMEM:F12 medium prior to measurement of *de novo* lipogenesis. Cells were then incubated in fresh DMEM:F12 medium (containing 0.5 mM sodium pyruvate, 0.5 mM L-glutamine, 2.5 mM glucose) supplemented with 1% fatty acid-free BSA, and containing 0.5 μCi [^14^C]-acetate (PerkinElmer, Waltham, MA, USA) and 5 μM sodium acetate for 12 hours at either 37°C or 31°C. At the end of the indicated incubation time, adipocytes were harvested, and lipids were extracted for analyses by thin layer chromatography.

### β-oxidation assay

Cultured adipocytes were washed 1× with PBS and then incubated with DMEM (following manufacturer’s guidelines; catalog #D5030; Sigma-Aldrich) containing BSA-conjugated approximately 3 μCi/ml of either [9,10-3H(N)]-palmitic acid, [9,10-3H(N)]-oleic Acid (PerkinElmer) or n-[2,2′,3,3′-3H] octanoic acid (American Radiolabeled Chemicals, St. Louis, MO, USA) and 22 μM of unlabeled NEFAs, respectively (Sigma-Aldrich). For long-chain fatty acid oxidation, assay media was contained with 200 μM L-carnitine (Sigma-Aldrich), and a subset of wells were treated with 40 μM etomoxir (Cayman Chemical) 2 hours before and duration of the assay. After 3 hours, conditioned media from cells was passed through columns containing AG1-X8 Anion Exchange Resin (Bio-Rad, Hercules, CA, USA) and collected in scintillation vials, then mixed with scintillation cocktail Bio-Safe II (RPI Research Product International, Mount Prospect, IL, USA). Radioactivity in the supernatant was measured using a scintillation counter.

#### Extracellular flux assay

Cellular and mitochondrial OCR were determined using the Seahorse XF96 Extracellular Flux Analyzer (Agilent, Santa Clara, CA, USA). To measure OCR on cool-adapted adipocytes, we used 2 different approaches and which gave similar results. In the first approach, a 96-well cell culture microplate for Seahorse was cut into half, and then approximately 480 MSCs were seeded into each well. Four days after seeding, adipogenesis was induced at 37°C as described above. Four days after induction of differentiation, adipocytes in half of the plate were cultured at 31°C, whereas the other half were incubated at 37°C for 12 days. At this point, the plate was rejoined in order to evaluate oxygen consumption at 31°C or at 37°C. In the second approach, differentiated adipocytes cultured at either 37°C or 31°C for 8 days (4 days before the assay) were trypsinized and isolated by centrifugation. Adipocytes were seeded on Cell-Tak–coated Seahorse XF96 microplates, then cultured overnight upside down at either 37°C or 31°C. One of the advantages of the first approach is that adipocytes are cultured on the same plates for the entire experiment. However, it is difficult in long-term culture to control cell numbers and thus basal OCR. Although we favor use of the second approach, and in our hands the replated adipocytes appear to maintain their differentiate morphology, one concern is the potential for adipocyte dedifferentiation. On the day of assay, the cells were washed and incubated with unbuffered XF assay medium (Agilent;103575) contained 5 mM glucose, 0.2 mM pyruvate, and 1 mM glutamine and placed the cell culture microplate into either 31°C or 37°C non-CO_2_ incubator for 1.5 hour prior to the assay. The sensor cartridge was loaded with oligomycin (Port A), FCCP (Port B), and rotenone/antimycin A (Port C) (XF Cell Mito Stress Test Kit, Agilent) to achieve final concentrations of 1 μM, 1 μM, and 0.5 μM, respectively. Other procedures for the assay were performed by following the manufacturer’s instructions. OCR was measured and normalized to DNA. Isolated mitochondria (5 μg protein) from MSC-derived adipocytes were seeded to XF96 microplates, and OCR was assayed as described above. Final concentrations of reagents are the following: 1 mM adenosine 5′-diphosphate (ADP), 10 mM succinate, 2 μM rotenone, 10 mM pyruvate, 1 mM malate, 40 μM palmitoylcarnitine, For the parametric equations: Basal respiration = (initial respiration)—(non-mitochondrial respiration); ATP-linked respiration = (last OCR before oligomycin)—(minimum OCR after oligomycin); Proton leak = (minimum OCR after oligomycin)—(non-mitochondrial OCR); Maximal respiration = (maximum rate after FCCP injection)—(non-mitochondrial respiration); SRC = (maximal respiration)—(basal respiration).

#### Isolation of mitochondria from adipose tissue and cultured MSC adipocytes

Isolation of mitochondria was as described previously [18]. Briefly, cultured MSC-derived adipocytes or adipocytes isolated from gluteal WAT were homogenized using a Potter-Elvehjem homogenizer and centrifuged at 800*g* for 10 minutes at 4°C. The supernatant was then centrifuged for 15 minutes at 8,000*g* at 4°C, and the pellet was washed with ice-cold buffer. After centrifugation at 7,000*g* for 10 minutes at 4°C, the pellet containing mitochondria was resuspended for analyses.

#### Metabolomics

Adipocytes from MSC were preincubated with fresh DMEM (#D5030; Sigma-Aldrich) with unlabeled glucose (5 mM), pyruvate (0.2 mM), and glutamine (1 mM) for 2 hours. For incubation in tracer-labeled media, a media change was performed. Substrate concentrations were kept constant except for the substitution of unlabeled metabolites with either ^13^C_6_ glucose (Sigma-Aldrich), 2,3-^13^C_2_ pyruvate (Cambridge Isotope Laboratories, Tewksbury, MA, USA), or ^13^C_5_ glutamate (Sigma-Aldrich) for 1 hour. Cells were then rapidly (<5 seconds) rinsed with 150 mM ammonium acetate and snap frozen by addition of liquid nitrogen directly to the cell plate. Metabolites from frozen cells were extracted by adding ice-cold 8:1:1 HPLC grade methanol:chloroform:water to the frozen cell plate, scraping to detach and lyse cells, transferring the suspension to microcentrifuge tubes, and centrifuging for 5 minutes at 15k RCF to remove pellet debris. Polar metabolites were analyzed in the supernatant by hydrophilic interaction chromatography-electrospray time of flight mass spectrometry (HILIC-ESI-TOF) as described previously [40].

#### Lipid extraction

Cultured adipocytes were washed twice with PBS and then suspended in 500 μl of a 1:2.5 methanol/water mixture. The cell suspension was transferred to a borosilicate glass tube. Wells were rinsed with 500 μl of the 1:2.5 methanol/water mixture, and the volume was transferred to a glass tube, then vortexed after adding 375 μl chloroform. Then, another 375 μl chloroform and 375 μl 0.9% NaCl were added to each tube, which was then vortexed vigorously and centrifuged at 2,500 rpm for 20 minutes at 4°C. The lower organic chloroform layer containing total lipids was transferred to a new tube and stored at −20°C until analyzed.

For the BMAT and WAT, tissue was transferred to a borosilicate glass tube after crushing on dry ice. Add 1,000 μl of the 1:2.5 methanol/water mixture 375 μl chloroform, then homogenize with immersion tissue grinder. Then, another 375 μl chloroform and 375 μl 0.9% NaCl were added to each tube, which was then vortexed vigorously and centrifuged at 2,500 rpm for 20 minutes at 4°C. The next step is the same as the process as described above for the cells.

#### Chromatographic separation of TAG and phospholipids

TAG and phospholipids were separated from the total lipid extract of cultured adipocytes and adipose tissue, by thin-layer chromatography applying the lipids as a band on a 20 × 20 cm silica gel thin-layer chromatography plate (silica gel 60, Millipore Sigma). The plate was developed with hexane-diethyl ether-acetic acid (80-20-1.5, v/v). The TAG and phospholipids were identified with respect to the retention flow (rf) of the authentic TAG and phospholipid standard applied on the same plate. TAG and phospholipid bands were scraped from the thin-layer chromatography plate, extracted with chloroform, and the lipids were subjected to derivatization as follows.

#### *Trans*-esterification with BF_3_-methanol and gas chromatography

Fatty acids in the extracted lipids were derivatized into their methyl esters by *trans*-esterification with boron trifluoride-methanol. The derivatized methyl esters were redissolved in a small volume of hexane and purified by thin-layer chromatography using n-hexane-diethyl ether-acetic acid (50:50:2, v/v/v) as the developing solvents. After development, plates were dried and sprayed with Premulin. Fatty acid methyl ester bands were identified under ultraviolet light by comparing the rf of methyl heptadecanoate (C17:0) standard (rf, 0.67) applied side by side on the same plate. Methyl esters were extracted from thin-layer chromatography powder with diethyl ether concentrated under nitrogen and redissolved in 100 μl hexane. The lipids’ fatty acid compositions were analyzed by gas chromatography (GC) as follows. FAMEs were analyzed with 1 μl sample injection on an Agilent GC machine, model 6890N equipped with a flame ionization detector, an autosampler model 7693 and a ChemStation software for data analysis. An Agilent HP 88 with a 30-m GC column with a 0.25-mm inner diameter and 0.20-mm film thickness was used. Hydrogen was used as a carrier gas, and nitrogen was used as a makeup gas. The analyses were conducted with a temperature programming of 125 to 220°C. The fatty acid components within unknown samples were identified with respect to the retention times of authentic standard methyl ester mixtures run side by side. The fatty acid components were quantified with respect to the known amount of internal standard added, and the calibration ratio was derived from each fatty acid of a standard methyl ester mixture and methyl heptadecanoate internal standard. The coefficient of variation for GC analyses was within 2.3% to 3.7%.

#### Lipidomics of adipocytes treated with SCD inhibitor

Cell plates were rapidly frozen with liquid nitrogen and maintained at −80°C until extraction. Cell plates were extracted on dry ice. To each well, 200 μL of ice cold methanol was added, and cells were scraped using a plastic cell scraper, then sample was transferred to a 1.5-mL microcentrifuge tube. The cell culture well was then rinsed with 658 μL of methyl tert-butyl ether (MTBE), and this solvent was added to the same 1.5-mL microcentrifuge tube. The samples were then sonicated for 5 minutes using a program of 20 seconds on, 10 seconds off, and amplitude of 30 (Qsonica (Newtown, CT, USA), chilled bath sonicator), temperature was maintained at 14°C. To induce phase separation, 164 μL water was added, and the solution was vortexed again for 10 seconds, then the sample was centrifuged for 2 minutes at 14,000*g* and 4°C. Then, 100 μL of the upper lipophilic layer was transferred to an amber glass autosampler vial with fused glass insert. This extract was dried by vacuum centrifugation for approximately 1 hour. Samples were resuspended in 50 μL of 9:1 methanol:toluene for analysis by LC–MS.

Sample analysis was performed using a Vanquish Binary Pump coupled to a Q-Exactive HF mass spectrometer (Thermo Fisher Scientific) as published previously [41,42]. For each sample, 10 μL of extract was chromatographically separated on an Acquity CSH C18 column (100 mm × 2.1 mm × 1.7 μm particle size; Waters, Milford, MA, USA) with a 400-μL/min flow rate and the column compartment held at 50°C. Mobile phases were prepared using Optima grade solvents: A consisted of 70% Acetonitrile with 10 mM ammonium acetate and 250 μL/L acetic acid; B consisted of 90:10 Isopropanol:Acetonitrile (v/v) with 10 mM ammonium acetate and 250 μL/L acetic acid. The following gradient was applied: 2% mobile phase B for 2 minutes, ramp up to 30% mobile phase B for 3 minutes, ramp to 50% mobile phase B for 1 minute, ramp to 85% mobile phase B for 14 minutes, ramp to 99% mobile phase B for 1 minute, and hold at 99% for 7 minutes. Reequilibrated with mobile phase B at 2% occurred for 1.75 minute.

Eluent was introduced onto the mass analyzer using a HESI II ESI source operating at 275°C, with 30 units of sheath gas, 6 units of aux gas (300°C), |4.0 kV| spray voltage (positive mode and negative mode), and 60 units of S-lens RF. Full MS and top2 MS2 spectra were collected in alternating polarity; full MS 200 to 1,600 m/z were collected at 30,000 resolution with 1 × 10^6^ automatic gain control (AGC) target, 100 ms ion accumulation time (max IT). MS2 scans were collected using 1 m/z isolation window, stepped normalized collision energy [20,30,40], and acquired at 30,000 resolution with 1 × 10^5^ AGC target and 50 ms max IT. A 30.0-second dynamic exclusion was employed.

Raw files were processed using Compound Discoverer 3.0 (Thermo Fisher Scientific) and LipiDex [42].

### qPCR to quantify mtDNA

DNA was prepared from cells using Gentra Puregene Kits including RNase A treatment (Qiagen, Germantown, MD, USA). mtDNA copy number per nuclear genome in MSC-derived adipocytes was quantified as described previously [18]. Primer sequences for PCR are in S1 Table.

### mRNA quantification by RT-PCR

RNA isolation, reverse transcription, and quantitative PCR were performed as previously described [18]. Primer sequences for real-time RT-PCR are in S1 Table.

#### RNA sequencing

Total RNA was isolated and purified as described above. After DNase treatment, samples were submitted to the University of Michigan Advanced Genomics Core for quality control, library preparation, and sequencing using the Illumina Hi-Seq platform. Read files were downloaded and concentrated into a single fastq file for each sample. Quality of raw read data was checked using FastQC (version v0.11.30) to identify features of the data that may indicate quality problems (i.e., low-quality scores, overrepresented sequences, and inappropriate GC content). The Tuxedo Suite software package was used for alignment, differential expression analysis, and post-analysis diagnostics. Briefly, reads were aligned to the UCSC reference genome using TopHat (version 2.0.13) and Bowtie2 (version 2.2.1). FastQC was used for the second round of quality control post-alignment to ensure that only high-quality data were input to expression quantitation and differential expression analysis. Differential expression analysis was done using 2 distinct methods to check for analysis consistency: Cufflinks/CuffDiff and HTSeq/DESeq2, using UCSC build mm10 as the reference genome sequence. A similar approach was taken to analyze the RNA-seq data for the *in vivo* samples, although alignment was conducted using STAR (version 2.7.5a), and differential expression analysis was conducted using featureCounts/DESeq2. Plots were generated using variations or alternative representations of native DESeq2 plotting functions, ggplot2, plotly, and other packages within the R environment. The accession number for the raw sequencing data for cultured adipocytes is NCBI Gene Expression Omnibus: GSE159451 and for gluteal WAT is GSE169142.

#### Pathway analysis

Pathway analysis was conducted on ranked lists of log2 fold change using GSEA v4.0.3 by the Broad Institute. Prior to analyses, mouse gene symbols were remapped to human ortholog symbols using chip annotation files. The mouse genes that did not have equivalent human orthologs were excluded from the analysis. The output from DESeq2 analysis was used to generate a list of genes ranked by the metric −log10 false discovery rate (FDR) * log2 fold change. The resulting list was run through preranked GSEA using the Molecular Signatures Database v7.1 (H, hallmark gene sets). Enriched pathways were defined by an FDR < 0.05. The normalized enrichment score (NES) is the primary metric from GSEA for evaluating the magnitude of differentially expressed pathways. Pathway impact analysis was conducted on AdvaitaBio’s iPathwayGuide to identify enriched KEGG pathways. The ggplot2 package in R was used for further visualization of the enriched pathways. Further pathway analyses were performed with Enrichr for genes regulated by cool temperatures in cultured cells and adipose tissue.

### Immunoblot

After lysis in 1% NP-40, 120 mM NaCl, 50 mM Tris-HCl (pH 7.4), 50 mM NaF, 2 mM EDTA, 1× protease inhibitor cocktail (Sigma-Aldrich), protein concentrations of lysates after centrifugation were measured by BCA protein assay (Thermo Fisher Scientific). Lysates were diluted to equal protein concentrations in lysis buffer and then boiled in SDS sample buffer (20 mM Tris (pH 6.8), 2% SDS, 0.01% bromophenol blue, 10% glycerol, 5% 2-mercaptoethanol) and subjected to SDS-PAGE and immunoblotting according to standard techniques. Separation of phosphorylated proteins using the Phos-Tag gel (FUJIFILM Wako Chemicals, Richmond, VA, USA) was according to the manufacturer’s instructions. Quantification of protein expression was done using ImageJ software. Antibodies used were as follows: ACC (Cell Signaling Technology, Danvers, MA, USA #3662), p-ACC(Ser79) (Cell Signaling Technology #3661), Adiponectin (Sigma Aldrich A6354), ACAA2 (MCKAT) (Thermo Fisher Scientific A21984), ATGL (Cell Signaling Technology #2138), β-actin (Cell Signaling Technology #4970), CPT1A (Abcam ab128568), CHREBP (Novus Biologicals, Centennial, CO, USA NB400-135), ERK2 (Santa Cruz Biotechnology, Dallas, TX, USA sc-1647), FASN (Abcam ab22759), FABP4 (R&D #1443), HSL (Cell Signaling Technology #4107), p-HSL (Ser563) (Cell Signaling Technology #4139), p-HSL (Ser660) (Cell Signaling Technology #4126). HADHB (MTPβ) (Novus Biologicals NBP1-54750), HSP70 (BD Transduction Lab, San Jose, CA, USA 610607), HSP90 (BD Transduction Lab 610418), Laminin (Novus Biologicals, NB300-144), OXPHOS Cocktail (Abcam ab110413), Perilipin (Abcam Ab3526), PMP70 (Thermo Fisher Scientific PA1650), PEX5 (Thermo Fisher Scientific PA558716), PPARg (Millipore MAB3872), SCD1 (Cell Signaling Technology #2438), SREBP1 (ThermoFisher Invitrogen 2A4), Tubulin (Invitrogen MA1-80017), UCP1 (Alpha Diagnostic, San Antonio, TX, USA UCP11-A), and VDAC1 (Abcam ab15895).

### Statistics

All data are presented as mean ± SD. When comparing 2 groups, significance was determined using 2-tailed Student *t* test. When comparing multiple experimental groups, an analysis of variance (ANOVA) was followed by post hoc analyses with Dunnett or Sidak test, as appropriate. Differences were considered significant at *p* < 0.05 and are indicated with asterisks. For metabolomics data analyses, the proportion of labeling at each carbon position was calculated by dividing each species by total sum of peak areas of all labeled positions. The proportion of data is well known to follow a beta distribution. Beta regression model is an extension of the generalized linear model with an assumption that the response variable follows a beta distribution with values in standard unit interval (0,1) [43]. Our flux proportion data contains 0 and 1 values depending on the labeling. Therefore, our response variable does not fall under the standard unit interval (0,1). Ospina and Ferrari proposed a more general class of zero-or-one inflated beta regression models for continuous proportions [44]. We proposed a zero-and-one inflated model, which was applied using R package GAMLSS [45]. To apply multiple testing correction, the significance is measured by a q-value calculated using the algorithm developed by Storey and Tibshirani [46]. All data analysis was performed in R.

## Supporting information

S1 Fig (**A**) Rats were housed from birth to 11 weeks of age at room temperature (22°C) or thermoneutrality (29°C). Lipid composition of phospholipids for pTib and dTib, and CV8 was determined by GC (*n* = 6). Desaturation index at the top of graph is (16:1 + 18:1)/(16:0 + 18:0). (**B, C**) Mice were housed from birth to 13 weeks either at 22°C or at 29°C without posterior hair after weaning. Whereas *Scd1* mRNA expression is elevated in subcutaneous WAT depots of mice at 22°C **(B**), *Adipoq* expression was not altered (**C**). Gene expression was normalized to geometric mean value of *Hprt*, *Tbp*, *Gapdh*, and *Ppia* and was expressed relative to 37°C control (*n* = 8–9). For panels (**A**–**C**), values are mean ± SD. *p < 0.05. Data shown are representative of at least 3 independent experiments. CV8, caudal vertebra-8; dTib, distal tibia; GC, gas chromatography; pTib, proximal tibia; SCD1, stearoyl-CoA desaturase-1; SFAs, saturated fatty acids; UFAs, unsaturated fatty acids; WAT, white adipose tissue.
(PDF)

S2 Fig (**A**) Cultured MSC adipocytes adapt to 31°C without morphological changes. Four days after differentiation, adipocytes were moved to the indicated temperatures for 12 days. (**B**) Adipocyte markers are expressed similarly between control cells cultured at 37°C and those incubated at 37°C from days 4 to 16. For adipocyte samples, 11 μg of protein lysate was analyzed per lane, whereas for brown adipose tissue, 0.1 or 0.5 μg lysate was included as a positive control for UCP1. (**C**) Beige adipocyte markers were not induced by exposure of cultured adipocytes to 31°C for the indicated days (*n* = 6). Gene expression was normalized to geometric mean value of *Hprt*, *Tbp*, and *Ppia* and was expressed relative to 37°C control (31°C for 0 day) (*n* = 4). * indicates significance at *p* < 0.05. Fabp4, fatty acid-binding protein 4; Fgf21, fibroblast growth factor 21; MSC, mesenchymal stem cell; PPARγ, peroxisome proliferator–activated receptor gamma; Ppargc1a, peroxisome proliferator–activated receptor gamma coactivator 1 alpha; UCP1, uncoupling protein 1.
(PDF)

S3 Fig RNA from mature adipocytes at days 0, 1, and 12 of cool adaptation was purified and subjected to RNA-seq analyses (*n =* 5 per time point). (**A**) MA plot showing the log2-mean expression versus log2-fold change of mRNA transcript expression in 12-day cool exposed MSC adipocytes compared to day 0. Each dot represents a gene. Twelve days of cool temperature exposure induced 1,872 genes (red) and suppressed 2,511 genes (blue). Significance was defined by an FDR <0.05 and absolute fold change >1.5. (**B**) Heat map of top 50 enriched and top 50 depleted genes in 12-day cool exposed MSC adipocytes. Color key based on rlog-transformed read count values and significance was defined by an FDR <0.01. FDR, false discovery rate; MSC, mesenchymal stem cell; NS, not significant; RNA-seq, RNA sequencing.
(PDF)

S4 Fig (**A**) Release of tritiated water from adipocytes treated with labeled octanoic acid. Adipocytes cultured at the indicated temperature for 12 days were incubated with tritiated octanoic acid for 3 hours in the presence and absence of etomoxir (*n* = 6). (**B, C**) Cool adaptation increases enzymes involved in synthesis and degradation of NEFAs. Lysates were collected after the indicated days of cool adaptation. SVCs from human (**B**) or eWAT from C57BL/6J mice **(C)** were differentiated into adipocytes. Human white preadipocytes (kindly provided by Dr. Shingo Kajimura; UCSF). (**D**) OXPHOS genes are up-regulated at the mRNA level. Heat map of genes involved in complexes I, II, III, VI, and V were constructed from KEGG map of OXPHOS genes (mmu00190). CPT1ɑ, carnitine palmitoyltransferase 1 alpha; eWAT, epididymal white adipose tissue; FASN, fatty acid synthase; NEFA, nonesterified fatty acid; OXPHOS, oxidative phosphorylation; SCD1, stearoyl-CoA desaturase-1; SVC, stromal vascular cell.
(PDF)

S5 Fig (**A**) Expression of UCP1 in adipocytes adapted to 31°C for the indicated days. A total of 11 μg of adipocyte or 0.5 or 1 μg BAT lysate was evaluated by immunoblot for UCP1. (**B**) Primary adipocytes isolated from eWAT or sWAT by collagenase digestion were cultured floating at either 37°C or 31°C for 2 days. BAT lysate (0.2, 0.5, or 1 μg) was used as a positive control for UCP1. (**C**) Cool adaptation increases enzymes involved in synthesis and degradation of NEFAs in adipocytes derived from UCP1 knockout mice. (**D**) Elevated basal OCR of adipocytes at 31°C is UCP1 independent. MSC adipocytes derived from WT or UCP1 KO mice were cultured at 31°C or 37°C for 12 days and basal OCR evaluated (*n* = 8). BAT, brown adipose tissue; CPT1ɑ, carnitine palmitoyltransferase 1 alpha; eWAT, epididymal white adipose tissue; FASN, fatty acid synthase; KO, knockout; MSC, mesenchymal stem cell; NEFA, nonesterified fatty acid; OCR, oxygen consumption rate; PPARγ, peroxisome proliferator–activated receptor gamma; SCD1, stearoyl-CoA desaturase-1; sWAT, subcutaneous white adipose tissue; UCP1, uncoupling protein 1; WT, wild-type.
(PDF)

S6 Fig (**A, B**) Cool adaptation increases the expression of HSL and ATGL but decreases phosphorylation of perilipin and HSL in response to CL-316,243. Mature adipocytes were adapted to 31°C for 12 days or remained at 37°C. Adipocytes were treated with (**A**) vehicle, 1 μM or 10 μM forskolin, or (**B**) vehicle, 0.05 μM, 0.2 μM, or 10 μM CL-316,243 for 30 minutes. Lysates were collected for immunoblot analyses. (**C**) Induction of enzymes involved in *de novo* lipogenesis with adaptation to 31°C is independent of ChREBP. Adipocytes derived from WT or ChREBP KO mice were cultured at 31°C or 37°C for 4 days before collecting samples. Although ChREBP deficiency impairs adipocytes differentiation, addition of rosiglitazone to differentiation media resulted in robust adipogenesis of both sets of cells, as has been reported. (**D**) On day 6 of MSC differentiation, adipocytes were infected in serum-free medium with adeno-sh*LacZ* control or adeno-sh*Sreb1* to induce gene knockdown. Adipocytes were then allowed to recover following infection and were cultured at the indicated temperature from day 8 to day 12. ACC, acetyl-CoA carboxylase; ATGL, adipose triglyceride lipase; ChREBP, carbohydrate response element-binding protein; Fabp4, fatty acid-binding protein 4; FASN, fatty acid synthase; HSL, hormone-sensitive lipase; KO, knockout; MSC, mesenchymal stem cell; SCD1, stearoyl-CoA desaturase-1; WT, wild-type.
(PDF)

S7 Fig Differentiated adipocytes cultured at either 37°C or 31°C for 8 days were trypsinized, then collected floating adipocytes following centrifugation. Adipocytes were seeded on Cell-Tak–coated Seahorse XF96 Cell Culture Microplate, then cultured overnight upside down in order to attach to the plate at either 37°C or 31°C. Adipocytes were cultured with SCD inhibitors: A-939572, MF-438, or CAY10566 at the indicated temperature for 2 days before the assay. (**A**) No effect of SCD inhibitors on SRC of adipocytes cultured at 37°C (*n* =  8–16 per group). (**B**) Increased SRC of adipocytes adapted to 31°C was inhibited with SCD inhibitors: A-939572, MF-438, or CAY10566 (*n* = 8–16 per group). FCCP, carbonyl cyanide-p-trifluoromethoxyphenylhydrazone; OCR, oxygen consumption rate; Oligo, oligomycin; Rot/Anti, rotenone and antimycin; SCD, stearoyl-CoA desaturase.
(PDF)

S1 Table qPCR primers and reagents used in this paper. (PDF)

S1 Data Numerical raw data. All numerical raw data associated with Fig 1 and S1 Fig. The file contains multiple tabs with labels corresponding to the relevant figure.
(XLSX)

S2 Data Numerical raw data. All numerical raw data associated with Fig 2 and S2 Fig. The file contains multiple tabs with labels corresponding to the relevant figure.
(XLSX)

S3 Data Numerical raw data. All numerical raw data associated with Fig 3 and S3 Fig. The file contains multiple tabs with labels corresponding to the relevant figure.
(XLSX)

S4 Data Numerical raw data. All numerical raw data associated with Fig 4 and S4 Fig. The file contains multiple tabs with labels corresponding to the relevant figure.
(XLSX)

S5 Data Numerical raw data. All numerical raw data associated with Fig 5 and S5 Fig. The file contains multiple tabs with labels corresponding to the relevant figure.
(XLSX)

S6 Data Numerical raw data. All numerical raw data associated with Fig 6. The file contains multiple tabs with labels corresponding to the relevant figure.
(XLSX)

S7 Data Numerical raw data. All numerical raw data associated with Fig 7 and S7 Fig. The file contains multiple tabs with labels corresponding to the relevant figure.
(XLSX)

S1 Raw Images Uncropped western blots from all main and Supporting information figures. (PDF)

## Abbreviations

ACC: acetyl-CoA carboxylase
AGC: automatic gain control
ANOVA: analysis of variance
ATGL: adipose triglyceride lipase
β3-AR: β3-adrenergic receptor
BMAd: bone marrow adipocyte
BMAT: bone marrow adipose tissue
BSA: bovine serum albumin
ChREBP: carbohydrate response element-binding protein
CPT1α: carnitine palmitoyltransferase 1α
CV: caudal vertebrae
dTib: distal tibia
eWAT: epididymal white adipose tissue
FASN: fatty acid synthase
FDR: false discovery rate
GC: gas chromatography
GSEA: gene set enrichment analysis
HBSS: Hanks’ balanced salt solution
HILIC-ESI-TOF: hydrophilic interaction chromatography-electrospray time of flight
HSL: hormone-sensitive lipase
LC–MS: liquid chromatography–mass spectrometry
MCKAT: medium-chain 3-ketoacyl-CoA thiolase
MSC: mesenchymal stem cell
MTBE: methyl tert-butyl ether
mtDNA: mitochondrial DNA
MTPβ: mitochondrial trifunctional protein beta subunit
ncDNA: nuclear DNA
NEFA: nonesterified fatty acid
NES: normalized enrichment score
OCR: oxygen consumption rate
OXPHOS: oxidative phosphorylation
RNA-seq: RNA sequencing
SCD1: stearoyl-CoA desaturase-1
SREBP-1c: sterol regulatory element-binding protein-1c
SVC: stromal vascular cell
TAG: triacylglycerol
WAT: white adipose tissue
