# Supplementary material for: The molecular and metabolic program by which white adipocytes adapt to cool physiologic temperatures
Source: PLoS Biol. 2021 May 12;19(5):e3000988. doi: 10.1371/journal.pbio.3000988 (PMC8143427; doi:10.1371/journal.pbio.3000988)
Supplement: S1 Fig — (A) Rats were housed from birth to 11 weeks of age at room temperature (22°C) or thermoneutrality (29°C). Lipid composition of phospholipids for pTib and dTib, and CV8 was determined by GC (n = 6). Desaturation index at the top of graph is (16:1 + 18:1)/(16:0 + 18:0). (B, C) Mice were housed from birth to 13 weeks either at 22°C or at 29°C without posterior hair after weaning. Whereas Scd1 mRNA expression is elevated in subcutaneous WAT depots of mice at 22°C (B), Adipoq expression was not altered (C). Gene expression was normalized to geometric mean value of Hprt, Tbp, Gapdh, and Ppia and was expressed relative to 37°C control (n = 8–9). For panels (A–C), values are mean ± SD. *p < 0.05. Data shown are representative of at least 3 independent experiments. CV8, caudal vertebra-8; dTib, distal tibia; GC, gas chromatography; pTib, proximal tibia; SCD1, stearoyl-CoA desaturase-1; SFAs, saturated fatty acids; UFAs, unsaturated fatty acids; WAT, white adipose tissue. (PDF) [file pbio.3000988.s001.pdf]

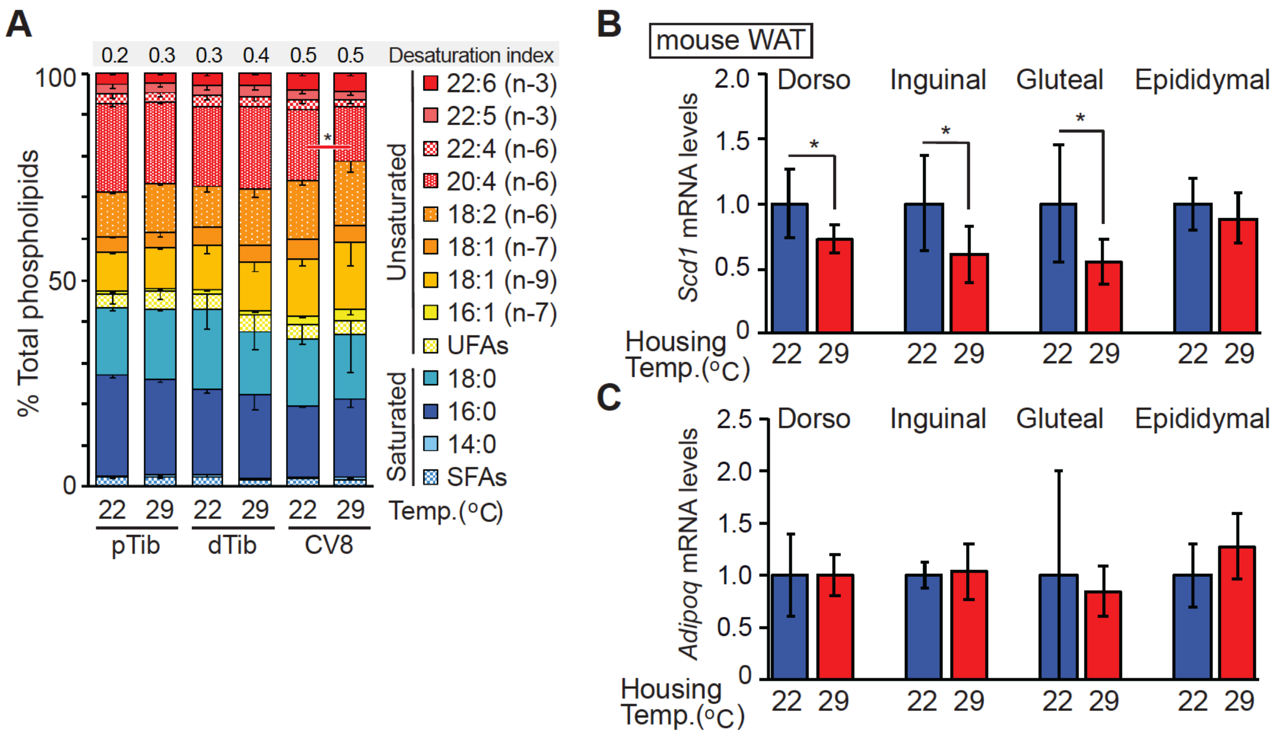

S1 Fig

(**A**) Rats were housed from birth to 11 weeks of age at room temperature (22°C) or thermoneutrality (29°C). Lipid composition of phospholipids for proximal (pTib) and distal tibia (dTib), and caudal vertebra-8 (CV8) was determined by gas chromatography (*n* = 6). Desaturation index at the top of graph is (16:1+18:1)/(16:0+18:0).

(**B** and **C**) Mice were housed from birth to 13 weeks either at 22°C or at 29°C without posterior hair after weaning. Whereas *Scd1* mRNA expression is elevated in subcutaneous WAT depots of mice at 22°C (**B**), *Adipoq* expression was not altered(**C**). Gene expression was normalized to geometric mean value of *Hprt*, *Tbp*, *Gapdh* and *Ppia,* and was expressed relative to 37°C control (*n* = 8-9). For panels (**A**-**C**), values are mean ± s.d. **p*<0.05. Data shown is representative of at least 3 independent experiments. Numerical data for all graphs are provided in S1 Data.
