# Supplementary material for: The molecular and metabolic program by which white adipocytes adapt to cool physiologic temperatures
Source: PLoS Biol. 2021 May 12;19(5):e3000988. doi: 10.1371/journal.pbio.3000988 (PMC8143427; doi:10.1371/journal.pbio.3000988)
Supplement: S2 Fig — (A) Cultured MSC adipocytes adapt to 31°C without morphological changes. Four days after differentiation, adipocytes were moved to the indicated temperatures for 12 days. (B) Adipocyte markers are expressed similarly between control cells cultured at 37°C and those incubated at 37°C from days 4 to 16. For adipocyte samples, 11 μg of protein lysate was analyzed per lane, whereas for brown adipose tissue, 0.1 or 0.5 μg lysate was included as a positive control for UCP1. (C) Beige adipocyte markers were not induced by exposure of cultured adipocytes to 31°C for the indicated days (n = 6). Gene expression was normalized to geometric mean value of Hprt, Tbp, and Ppia and was expressed relative to 37°C control (31°C for 0 day) (n = 4). * indicates significance at p < 0.05. Fabp4, fatty acid-binding protein 4; Fgf21, fibroblast growth factor 21; MSC, mesenchymal stem cell; PPARγ, peroxisome proliferator–activated receptor gamma; Ppargc1a, peroxisome proliferator–activated receptor gamma coactivator 1 alpha; UCP1, uncoupling protein 1. (PDF) [file pbio.3000988.s002.pdf]

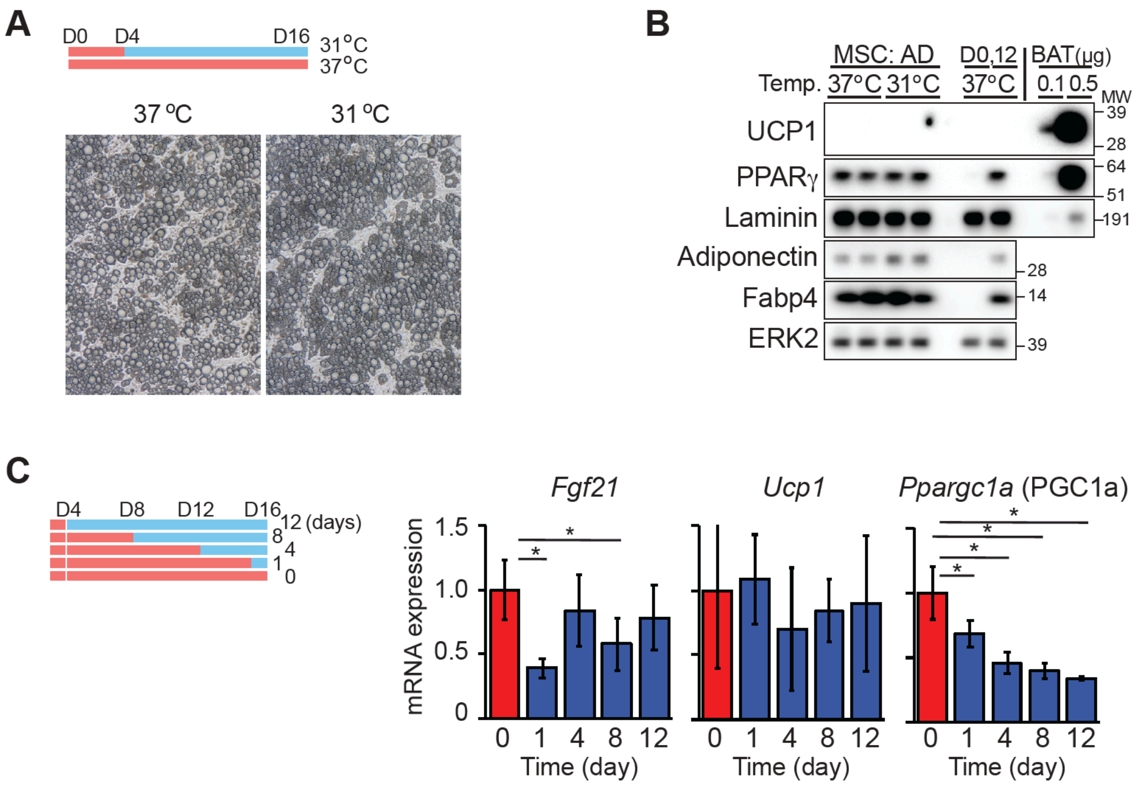

S2 Fig

(**A**) Cultured MSC adipocytes adapt to 31°C without morphological changes. Four days after differentiation, adipocytes were moved to the indicated temperatures for 12 days.

(**B**) Adipocyte markers are expressed similarly between control cells cultured at 37°C and those incubated at 37°C from day 4 to 16. For adipocyte samples, 11 μg of protein lysate was analyzed per lane, whereas for brown adipose tissue, 0.1 or 0.5 μg lysate was included as a positive control for UCP1.

(**C**) Beige adipocyte markers were not induced by exposure of cultured adipocytes to 31°C for the indicated days ($n = 6$). Gene expression was normalized to geometric mean value of *Hprt*, *Tbp*, *Ppia,* and was expressed relative to 37°C control (31°C for 0 day) ($n = 4$). * indicates significance at $p < 0.05$. Uncropped western blots are provided in S8 Raw Images, and numerical data for all graphs are provided in S2 Data.
