# Supplementary material for: The molecular and metabolic program by which white adipocytes adapt to cool physiologic temperatures
Source: PLoS Biol. 2021 May 12;19(5):e3000988. doi: 10.1371/journal.pbio.3000988 (PMC8143427; doi:10.1371/journal.pbio.3000988)
Supplement: S3 Fig — (A) MA plot showing the log2-mean expression versus log2-fold change of mRNA transcript expression in 12-day cool exposed MSC adipocytes compared to day 0. Each dot represents a gene. Twelve days of cool temperature exposure induced 1,872 genes (red) and suppressed 2,511 genes (blue). Significance was defined by an FDR <0.05 and absolute fold change >1.5. (B) Heat map of top 50 enriched and top 50 depleted genes in 12-day cool exposed MSC adipocytes. Color key based on rlog-transformed read count values and significance was defined by an FDR <0.01. FDR, false discovery rate; MSC, mesenchymal stem cell; NS, not significant; RNA-seq, RNA sequencing. (PDF) [file pbio.3000988.s003.pdf]

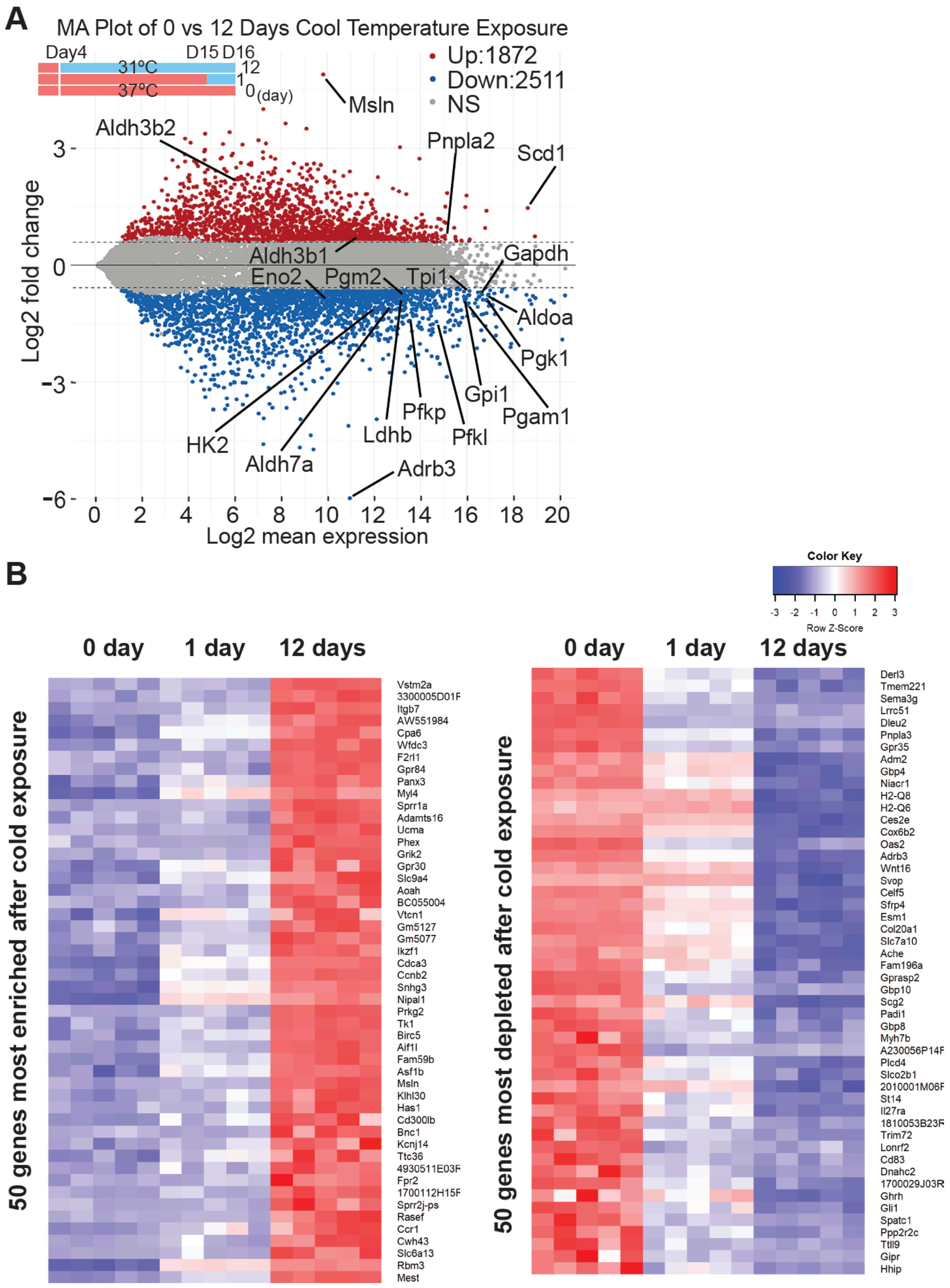
A
MA Plot of 0 vs 12 Days Cool Temperature Exposure
Day4
D15 D16
31°C
37°C
12
1
0 (day)
Up:1872
Down:2511
NS
Msln
Aldh3b2
Pnpla2
Scd1
Aldh3b1
Gapdh
Eno2
Pgm2
Tpi1
Aldoa
Pgk1
Gpi1
Pgam1
Pfkp
Ldhb
Pfkl
HK2
Aldh7a
Adrb3
Log2 fold change
Log2 mean expression
B
Color Key
Row Z-Score
0 day
1 day
12 days
50 genes most enriched after cold exposure
Vstm2a
3300005D01F
Itgb7
AW551984
Cpa6
Wfdc3
F2rl1
Gpr84
Panx3
Myl4
Sprr1a
Adamts16
Ucma
Phex
Grik2
Gpr30
Slc9a4
Aoah
BC055004
Vtcn1
Gm5127
Gm5077
Ikzf1
Cdca3
Ccnb2
Snhg3
Nipal1
Prkg2
Tk1
Birc5
Aif1l
Fam59b
Asf1b
Msln
Klhl30
Has1
Cd300lb
Bnc1
Kcnj14
Ttc36
4930511E03F
Fpr2
1700112H15F
Sprr2j-ps
Rasef
Ccr1
Cwh43
Slc6a13
Rbm3
Mest
50 genes most depleted after cold exposure
Derl3
Tmem221
Sema3g
Lrrc51
Dleu2
Pnpla3
Gpr35
Adm2
Gbp4
Niacr1
H2-Q8
H2-Q6
Ces2e
Cox6b2
Oas2
Adrb3
Wnt16
Svop
Celf5
Sfrp4
Esm1
Col20a1
Slc7a10
Ache
Fam196a
Gprasp2
Gbp10
Scg2
Padi1
Gbp8
Myh7b
A230056P14F
Plcd4
Slco2b1
2010001M06F
St14
Il27ra
1810053B23F
Trim72
Lonrf2
Cd83
Dnahc2
1700029J03R
Ghrh
Gli1
Spatc1
Ppp2r2c
Ttl9
Gipr
Hhip

S3 Fig

RNA from mature adipocytes at day 0, 1 and 12 of cool adaptation was purified and subjected to RNA-Seq analyses (n = 5 per time point).

(**A**) MA plot showing the log2-mean expression versus log2-fold change of mRNA transcript expression in 12-day cool exposed MSC adipocytes compared to day 0. Each dot represents a gene. Twelve days of cool temperature exposure induced 1872 genes (red) and suppressed 2511 genes (blue). Significance was defined by a FDR < 0.05 and absolute fold change > 1.5.

(**B**) Heat map of top 50 enriched and top 50 depleted genes in 12-day cool exposed MSC adipocytes. Color key based on rlog-transformed read count values and significance was defined by an FDR < 0.01. Numerical data for all graphs are provided in S3 Data.
