# Supplementary material for: The molecular and metabolic program by which white adipocytes adapt to cool physiologic temperatures
Source: PLoS Biol. 2021 May 12;19(5):e3000988. doi: 10.1371/journal.pbio.3000988 (PMC8143427; doi:10.1371/journal.pbio.3000988)
Supplement: S4 Fig — (A) Release of tritiated water from adipocytes treated with labeled octanoic acid. Adipocytes cultured at the indicated temperature for 12 days were incubated with tritiated octanoic acid for 3 hours in the presence and absence of etomoxir (n = 6). (B, C) Cool adaptation increases enzymes involved in synthesis and degradation of NEFAs. Lysates were collected after the indicated days of cool adaptation. SVCs from human (B) or eWAT from C57BL/6J mice (C) were differentiated into adipocytes. Human white preadipocytes (kindly provided by Dr. Shingo Kajimura; UCSF). (D) OXPHOS genes are up-regulated at the mRNA level. Heat map of genes involved in complexes I, II, III, VI, and V were constructed from KEGG map of OXPHOS genes (mmu00190). CPT1ɑ, carnitine palmitoyltransferase 1 alpha; eWAT, epididymal white adipose tissue; FASN, fatty acid synthase; NEFA, nonesterified fatty acid; OXPHOS, oxidative phosphorylation; SCD1, stearoyl-CoA desaturase-1; SVC, stromal vascular cell. (PDF) [file pbio.3000988.s004.pdf]

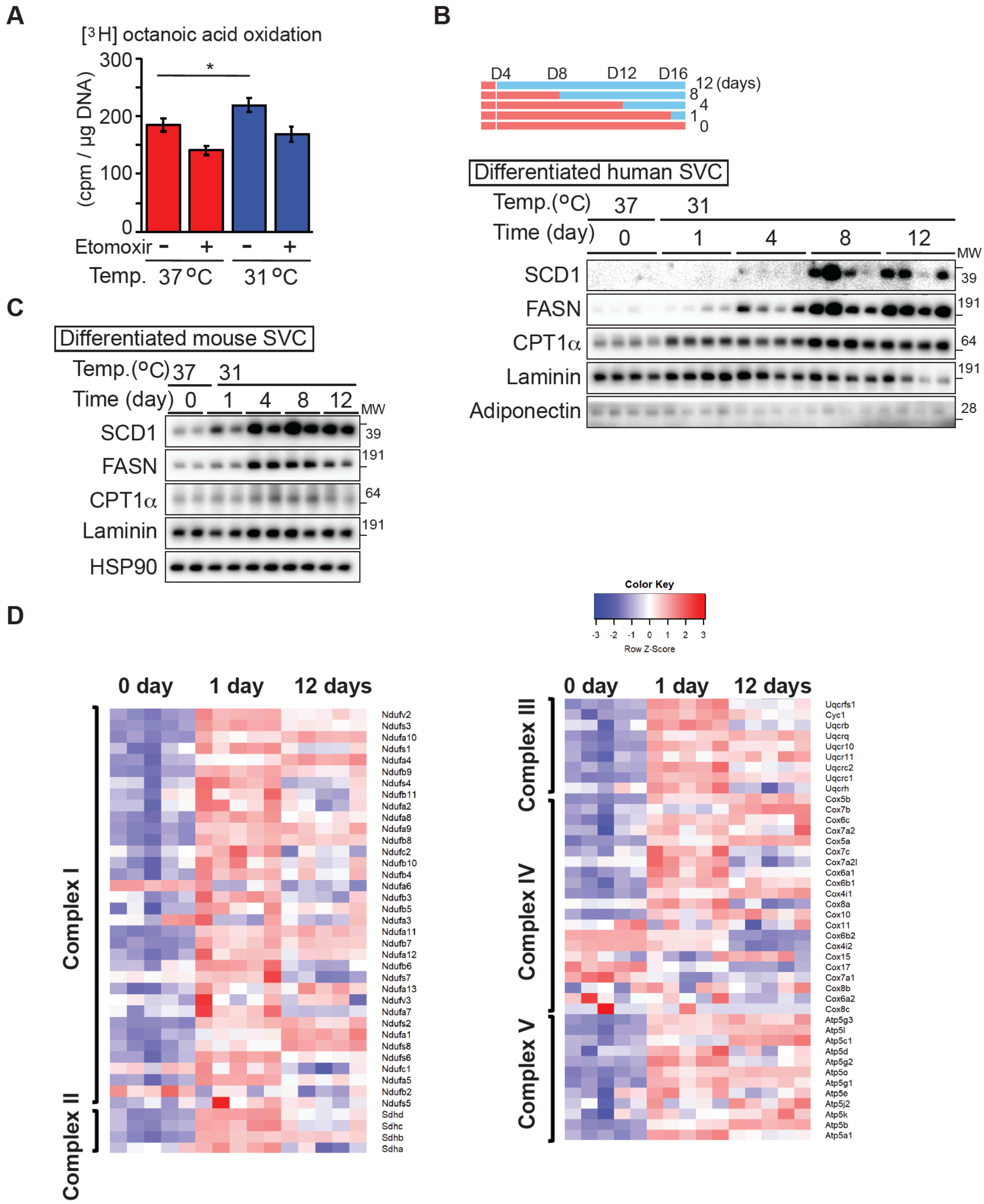
A
[3H] octanoic acid oxidation
(cpm / µg DNA)
300
200
100
0
*
Etomoxir - + - +
Temp. 37 °C 31 °C
B
D4 D8 D12 D16
12 (days)
8
4
1
0
Differentiated human SVC
Temp.(°C) 37 31
Time (day) 0 1 4 8 12
MW
SCD1
39
FASN
191
CPT1α
64
Laminin
191
Adiponectin
28
C
Differentiated mouse SVC
Temp.(°C) 37 31
Time (day) 0 1 4 8 12
MW
SCD1
39
FASN
191
CPT1α
64
Laminin
191
HSP90
D
Color Key
-3 -2 -1 0 1 2 3
Row Z-Score
0 day 1 day 12 days
Complex I
Complex II
Ndufv2
Ndufs3
Ndufa10
Ndufs1
Ndufa4
Ndufb9
Ndufs4
Ndufb11
Ndufa2
Ndufa8
Ndufa9
Ndufb8
Ndufc2
Ndufb10
Ndufb4
Ndufa6
Ndufb3
Ndufb5
Ndufa3
Ndufa11
Ndufb7
Ndufa12
Ndufb6
Ndufs7
Ndufa13
Ndufv3
Ndufa7
Ndufs2
Ndufa1
Ndufs8
Ndufs6
Ndufc1
Ndufa5
Ndufb2
Ndufs5
Sdhd
Sdhc
Sdhb
Sdha
0 day 1 day 12 days
Complex III
Complex IV
Complex V
Uqcrfs1
Cyc1
Uqcrb
Uqcrq
Uqcr10
Uqcr11
Uqcrc2
Uqcrc1
Uqcrh
Cox5b
Cox7b
Cox6c
Cox7a2
Cox5a
Cox7c
Cox7a2l
Cox6a1
Cox6b1
Cox4i1
Cox8a
Cox10
Cox11
Cox6b2
Cox4i2
Cox15
Cox17
Cox7a1
Cox8b
Cox6a2
Cox8c
Atp5g3
Atp5l
Atp5c1
Atp5d
Atp5g2
Atp5o
Atp5g1
Atp5e
Atp5j2
Atp5k
Atp5b
Atp5a1

S4 Fig

(**A**) Release of tritiated water from adipocytes treated with labeled octanoic acid. Adipocytes cultured at the indicated temperature for 12 days were incubated with tritiated octanoic acid for 3 hrs in the presence and absence of etomoxir ($n = 6$).

(**B** and **C**) Cool adaptation increases enzymes involved in synthesis and degradation of non-esterified fatty acids. Lysates were collected after the indicated days of cool adaptation. Stromal-vascular cells (SVC) of SVC from human (**B**) or eWAT from C57BL/6J mice **(C)** were differentiated into adipocytes. Human white preadipocytes (kindly provided by Dr. Shingo Kajimura; UCSF) were differentiated as previously described (1).

(**D**) Oxidative phosphorylation genes are upregulated at the mRNA level. Heat map of genes involved in complexes I, II, III, VI, and V were constructed from KEGG map of oxidative phosphorylation genes (mmu00190). Uncropped western blots are provided in S8 Raw Images, and numerical data for all graphs are provided in S4 Data.

1. Shinoda K, Luijten IH, Hasegawa Y, Hong H, Sonne SB, Kim M, et al. Genetic and functional characterization of clonally derived adult human brown adipocytes. Nat Med. 2015;21(4):389-94.
