# Supplementary material for: The molecular and metabolic program by which white adipocytes adapt to cool physiologic temperatures
Source: PLoS Biol. 2021 May 12;19(5):e3000988. doi: 10.1371/journal.pbio.3000988 (PMC8143427; doi:10.1371/journal.pbio.3000988)
Supplement: S5 Fig — (A) Expression of UCP1 in adipocytes adapted to 31°C for the indicated days. A total of 11 μg of adipocyte or 0.5 or 1 μg BAT lysate was evaluated by immunoblot for UCP1. (B) Primary adipocytes isolated from eWAT or sWAT by collagenase digestion were cultured floating at either 37°C or 31°C for 2 days. BAT lysate (0.2, 0.5, or 1 μg) was used as a positive control for UCP1. (C) Cool adaptation increases enzymes involved in synthesis and degradation of NEFAs in adipocytes derived from UCP1 knockout mice. (D) Elevated basal OCR of adipocytes at 31°C is UCP1 independent. MSC adipocytes derived from WT or UCP1 KO mice were cultured at 31°C or 37°C for 12 days and basal OCR evaluated (n = 8). BAT, brown adipose tissue; CPT1ɑ, carnitine palmitoyltransferase 1 alpha; eWAT, epididymal white adipose tissue; FASN, fatty acid synthase; KO, knockout; MSC, mesenchymal stem cell; NEFA, nonesterified fatty acid; OCR, oxygen consumption rate; PPARγ, peroxisome proliferator–activated receptor gamma; SCD1, stearoyl-CoA desaturase-1; sWAT, subcutaneous white adipose tissue; UCP1, uncoupling protein 1; WT, wild-type. (PDF) [file pbio.3000988.s005.pdf]

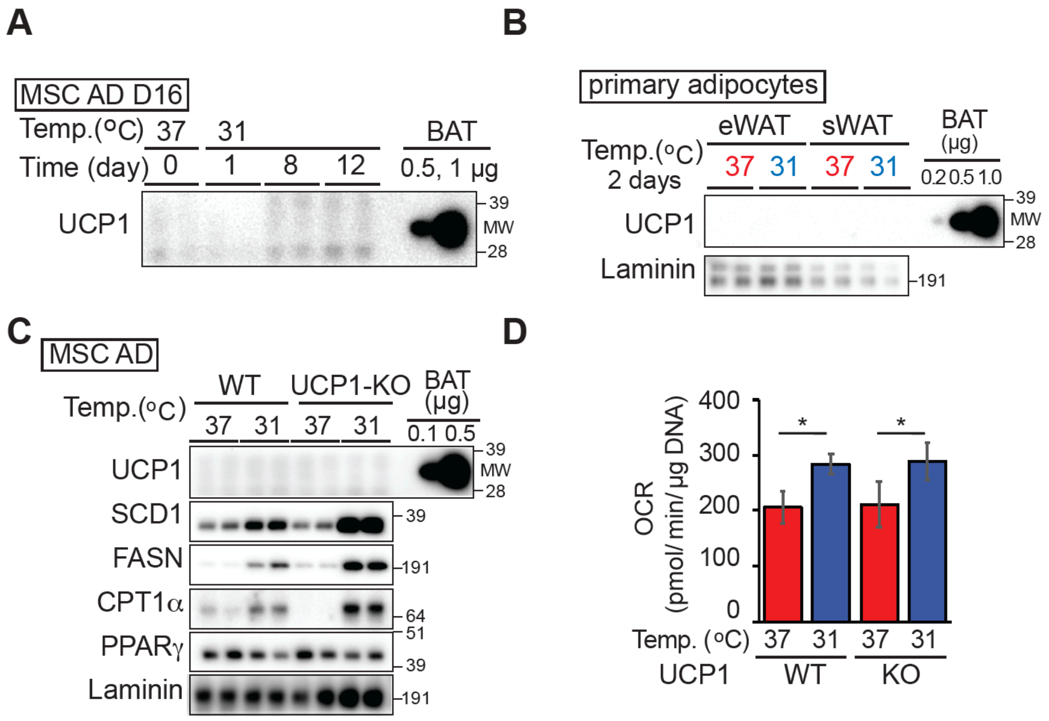

S5 Fig

(**A**) Expression of UCP1 in adipocytes adapted to 31°C for the indicated days. 11 µg of adipocyte or 0.5 or 1 µg BAT lysate was evaluated by immunoblot for UCP1.

(**B**) Primary adipocytes isolated from epididymal white adipose tissue (eWAT) or subcutaneous gluteal WAT (sWAT) by collagenase digestion were cultured floating at either 37°C or 31°C for 2 days. Brown adipose tissue lysate (0.2, 0.5 or 1 µg) was used as a positive control for UCP1.

(**C**) Cool adaptation increases enzymes involved in synthesis and degradation of non-esterified fatty acids in adipocytes derived from UCP1 knockout mice.

(**D**) Elevated basal OCR of adipocytes at 31°C is UCP1 independent. MSC adipocytes derived from wild type or UCP1 knockout mice were cultured at 31°C or 37°C for 12 days and basal OCR evaluated ($n = 8$). Uncropped western blots are provided in S8 Raw Images, and numerical data for all graphs are provided in S5 Data.
