# Supplementary material for: The molecular and metabolic program by which white adipocytes adapt to cool physiologic temperatures
Source: PLoS Biol. 2021 May 12;19(5):e3000988. doi: 10.1371/journal.pbio.3000988 (PMC8143427; doi:10.1371/journal.pbio.3000988)
Supplement: S6 Fig — (A, B) Cool adaptation increases the expression of HSL and ATGL but decreases phosphorylation of perilipin and HSL in response to CL-316,243. Mature adipocytes were adapted to 31°C for 12 days or remained at 37°C. Adipocytes were treated with (A) vehicle, 1 μM or 10 μM forskolin, or (B) vehicle, 0.05 μM, 0.2 μM, or 10 μM CL-316,243 for 30 minutes. Lysates were collected for immunoblot analyses. (C) Induction of enzymes involved in de novo lipogenesis with adaptation to 31°C is independent of ChREBP. Adipocytes derived from WT or ChREBP KO mice were cultured at 31°C or 37°C for 4 days before collecting samples. Although ChREBP deficiency impairs adipocytes differentiation, addition of rosiglitazone to differentiation media resulted in robust adipogenesis of both sets of cells, as has been reported. (D) On day 6 of MSC differentiation, adipocytes were infected in serum-free medium with adeno-shLacZ control or adeno-shSreb1 to induce gene knockdown. Adipocytes were then allowed to recover following infection and were cultured at the indicated temperature from day 8 to day 12. ACC, acetyl-CoA carboxylase; ATGL, adipose triglyceride lipase; ChREBP, carbohydrate response element-binding protein; Fabp4, fatty acid-binding protein 4; FASN, fatty acid synthase; HSL, hormone-sensitive lipase; KO, knockout; MSC, mesenchymal stem cell; SCD1, stearoyl-CoA desaturase-1; WT, wild-type. (PDF) [file pbio.3000988.s006.pdf]

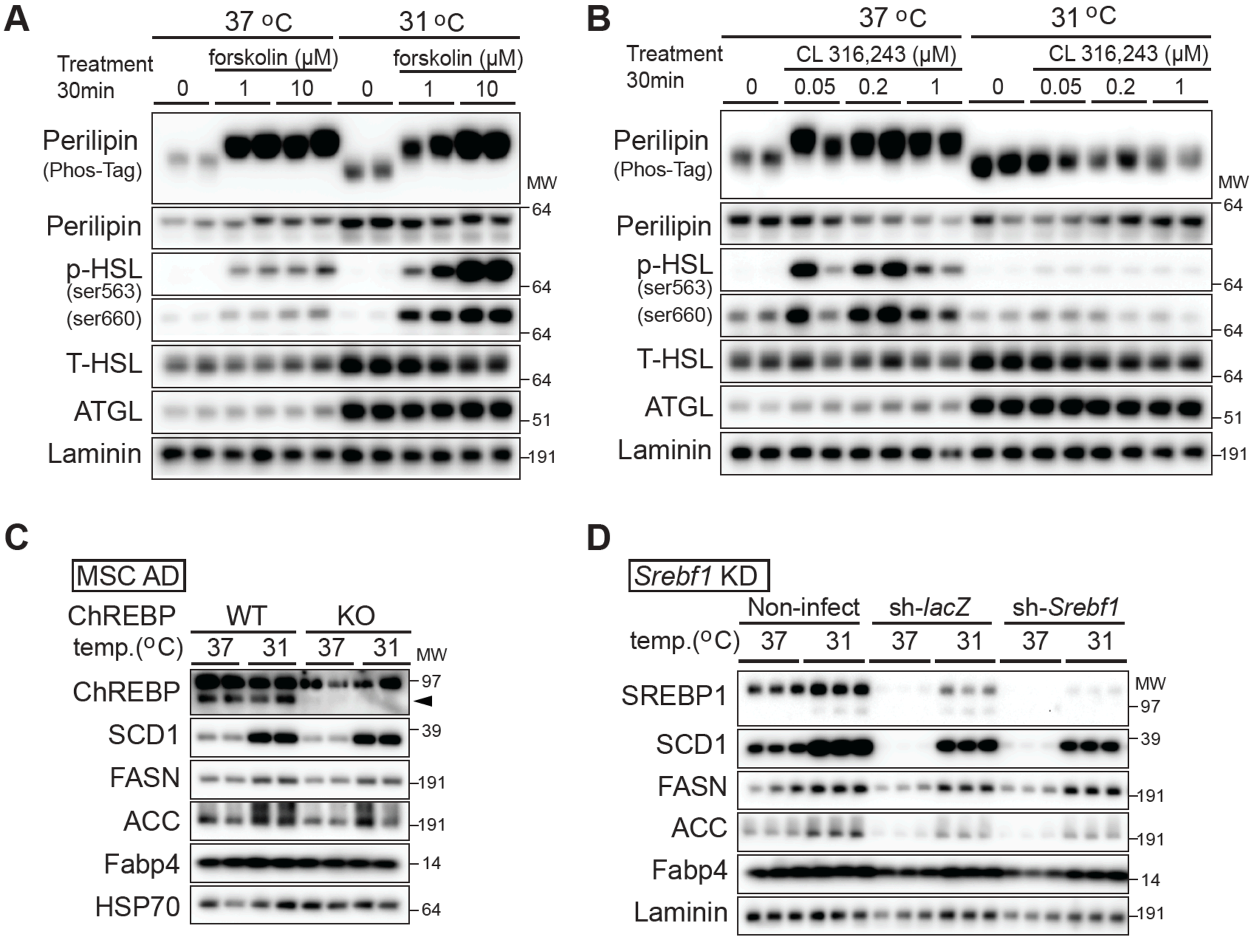

S6 Fig

(**A** and **B**) Cool adaptation increases the expression of HSL and ATGL but decreases phosphorylation of perilipin and HSL in response to CL-316,243. Mature adipocytes were adapted to 31°C for 12 days or remained at 37°C. Adipocytes were treated with **(A)** vehicle, 1 μM or 10 μM forskolin, or **(B)** vehicle, 0.05 μM, 0.2 μM or 10 μM CL-316,243 for 30 min. Lysates were collected for immunoblot analyses.

(**C**) Induction of enzymes involved in *de novo* lipogenesis with adaptation to 31°C is independent of ChREBP. Adipocytes derived from wildtype or ChREBP knockout mice were cultured at 31°C or 37°C for 4 days before collecting samples. Although ChREBP-deficiency impairs adipocytes differentiation (1), addition of rosiglitazone to differentiation media resulted in robust adipogenesis of both sets of cells, as has been reported (2).

(**D**) On day six of MSC differentiation, adipocytes were infected in serum-free medium with adeno-sh*LacZ* control or adeno-sh*Sreb1* to induce gene knockdown. Adipocytes were then allowed to recover following infection and were cultured at the indicated temperature from day 8 to day 12. Uncropped western blots are provided in S8 Raw Images.**S7 Fig**

1. Witte N, Muenzner M, Rietscher J, Knauer M, Heidenreich S, Nuotio-Antar AM, et al. The Glucose Sensor ChREBP Links De Novo Lipogenesis to PPARgamma Activity and Adipocyte Differentiation. Endocrinology. 2015;156(11):4008-19.
2. Vijayakumar A, Aryal P, Wen J, Syed I, Vazirani RP, Moraes-Vieira PM, et al. Absence of Carbohydrate Response Element Binding Protein in Adipocytes Causes Systemic Insulin Resistance and Impairs Glucose Transport. Cell Rep. 2017;21(4):1021-35.
