# Supplementary material for: The molecular and metabolic program by which white adipocytes adapt to cool physiologic temperatures
Source: PLoS Biol. 2021 May 12;19(5):e3000988. doi: 10.1371/journal.pbio.3000988 (PMC8143427; doi:10.1371/journal.pbio.3000988)
Supplement: S7 Fig — Adipocytes were seeded on Cell-Tak–coated Seahorse XF96 Cell Culture Microplate, then cultured overnight upside down in order to attach to the plate at either 37°C or 31°C. Adipocytes were cultured with SCD inhibitors: A-939572, MF-438, or CAY10566 at the indicated temperature for 2 days before the assay. (A) No effect of SCD inhibitors on SRC of adipocytes cultured at 37°C (n = 8–16 per group). (B) Increased SRC of adipocytes adapted to 31°C was inhibited with SCD inhibitors: A-939572, MF-438, or CAY10566 (n = 8–16 per group). FCCP, carbonyl cyanide-p-trifluoromethoxyphenylhydrazone; OCR, oxygen consumption rate; Oligo, oligomycin; Rot/Anti, rotenone and antimycin; SCD, stearoyl-CoA desaturase. (PDF) [file pbio.3000988.s007.pdf]

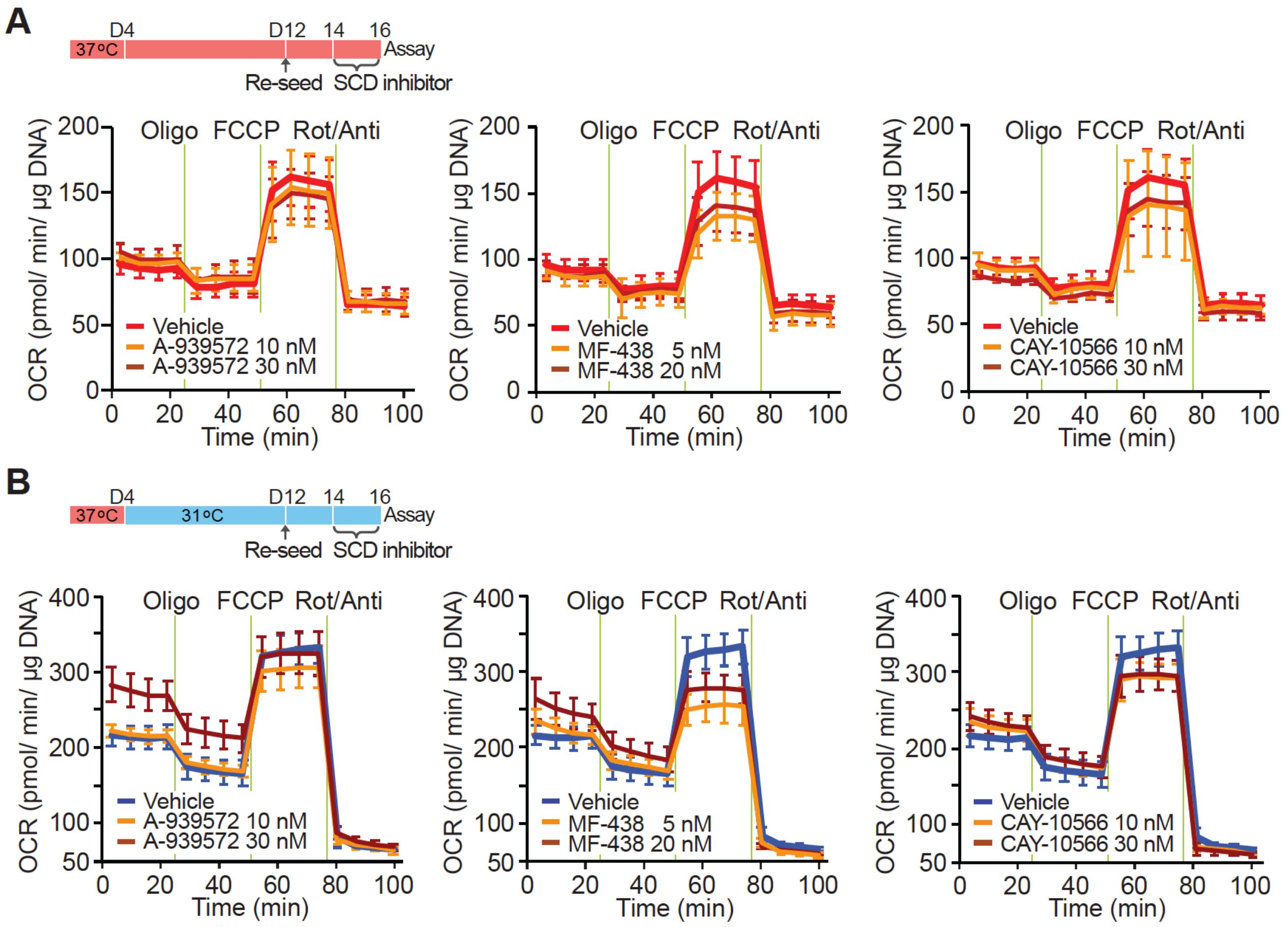

S7 Fig

Differentiated adipocytes cultured at either 37°C or 31°C for 8 days were trypsinized, then collected floating adipocytes following centrifugation. Adipocytes were seeded on Cell-Tak coated Seahorse XF96 Cell Culture Microplate, then cultured overnight upside down in order to attach to the plate at either 37°C or 31°C. Adipocytes were cultured with SCD inhibitors: A-939572, MF-438, or CAY10566 at the indicated temperature for two days before the assay. (**A**) No effect of SCD inhibitors on SRC of adipocytes cultured at 37°C (*n* = 8-16 per group). (**B**) Increased SRC of adipocytes adapted to 31°C was inhibited with SCD inhibitors; A-939572, MF-438, or CAY10566 (*n* = 8-16 per group). Uncropped western blots are provided in S8 Raw Images, and numerical data for all graphs are provided in S7 Data.
