# Supplementary material for: The molecular and metabolic program by which white adipocytes adapt to cool physiologic temperatures
Source: PLoS Biol. 2021 May 12;19(5):e3000988. doi: 10.1371/journal.pbio.3000988 (PMC8143427; doi:10.1371/journal.pbio.3000988)
Supplement: S1 Table — (PDF) [file pbio.3000988.s008.pdf]

S1 Table

qPCR primers used were as follows;

Gene name	Forward	Reverse
Adrb2	TACGTGCCCCTGGTGGTGA	AGGTTTTGGGCGTGGAATCTT
Adrb3	CAGGCGCCACACGAGATG	GCGGGCGATGGCTATGAT
Adipoq	AAGAAGGACAAGGCCGTTCTCTT	GCTATGGGTAGTTGCAGTCAGTT
B2m	CCGCCTCACATTGAAATCCA	TCGATCCCAGTAGACGGTCTTG
Gapdh	TGACGTGCCGCCTGGAGAAA	AGTGTAGCCCAAGATGCCCTTCAG
Hprt	TCATTATGCCGAGGATTTGGA	GCACACAGAGGGCCACAAT
Ppia	CACCGTGTTCTTCGACATCA	CAGTGCTCAGAGCTCGAAACT
Rn18s	CGCTTCCTTACCTGGTTGAT	GAGCGACCAAAGGAACCATA
Rpl13a	CCCTCCACCCTATGACAAGA	GCCCCAGGTAAGCAAACTT
Rpl32	GAGCAACAAGAAAACCAAGCA	TGCACACAAGCCATCTACTCA
Scd1	CGTGGGTTGGCTGCTTGTG	CAGGAGGCCGGGCTTGTAGT
Tbp	ACCTTATGCTCAGGGCTTGG	GCCGTAAGGCATCATTGGAC
mtDNA/nuc DNA		
CytoB	CATTTATTATCGCGGCCCTA	TGTTGGGTTGTTTGATCCTG
Cox1-Tms1	GCCTTTCAGGAATACCACGA	CCAATTTTAGGGGGTTCGAT
Glucagon	CAGGGCCATCTCAGAACC	GCTATTGGAAAGCCTCTTGC
β-Globin	GAAGCGATTCTAGGGAGCAG	GGAGCAGCGATTCTGAGTAGA

REAGENT or RESOURCE	SOURCE	IDENTIFIER.	
**Antibodies**			
			expected size; (kDa)
ACC	Cell Signaling Technology	#3662	280
p-ACC(Ser79)	Cell Signaling Technology	#3661	280
Adiponectin	Sigma Aldrich	A6354	30
ACAA2 (MCKAT)	Thermo Fisher Scientific	A21984	42
ATGL	Cell Signaling Technology	#2138	54
β-actin	Cell Signaling	#4970	45
CPT1A	Abcam	ab128568	88
CHREBP	Novus Biologicals	NB400-135	95
ERK 2	Santa Cruz Biotechnology	sc-1647	42
FASN	Abcam	ab22759	273
FABP4	R&D	#1443	15
HSL	Cell Signaling Technology	#4107	81/83
p-HSL (Ser563)	Cell Signaling Technology	#4139	81/83
p-HSL (Ser660)	Cell Signaling Technology	#4126	81/83
HADHB (MTPβ)	Novus Biologicals	NBP1-54750	47
HSP70	BD Transduction lab.	610607	72
HSP90	BD Transduction lab.	610418	90
Laminin	Novus Biologicals	NB300-144	220
OXPHOS Cocktail	Abcam	ab110413	55/48/40/30/22
Perilipin	Abcam	Ab3526	62
PMP70	Thermo Fisher Scientific	PA1650	70
PEX5	Thermo Fisher Scientific	PA558716	80
PPARg	Millipore	MAB3872	54
SCD1	Cell Signaling	#2438	37
SREBP1	ThermoFisher invitrogen	2A4	120
Tubulin	Invitrogen	MA1-80017	51
UCP1	Alpha Diagnostic	UCP11-A	30
VDAC1	Abcam	ab15895	32

REAGENT or RESOURCE	SOURCE	IDENTIFIER.
**Chemicals, Peptides, and Recombinant Proteins**		
adenosine 5'-diphosphate	Sigma-Aldrich	
A-939572	Cayman Chemical	No.19123
CAY10566	Cayman Chemical	
Cell-Tak	Corning	
CL-316,243	Tocris	
C75	Cayman Chemical	
etomoxir	Cayman Chemical	
forskolin	Tocris	

MF-438	Sigma-Aldrich	
octanoic acid	Sigma-Aldrich	
oleic acid	Sigma-Aldrich	
rotenone	Tocris	
palmitoleic acid	Sigma-Aldrich	
palmitoyl carnitine	Sigma-Aldrich	
pyruvate	Sigma-Aldrich	
collagenase type I	Worthington Biochemical	
fatty acid-free bovine serum albumin	Gold Biotechnology	
Rosiglitazone	Cayman Chemical	No. 71740
sodium palmitate	Sigma-Aldrich	
5-(tetradecyloxy)-2-furoic acid (TOFA)	Cayman Chemical	
AG1-X8 Anion Exchange Resin	Bio-Rad	
scintillation cocktail Bio-Safe II	RPI research product international	
TLC plate: silica gel 60	Millipore Sigma	
$^{13}C_6$ glucose	Sigma	
2,3-$^{13}C_2$ pyruvate	Cambridge Isotope	
$^{13}C_5$ glutamate	Sigma	
protease inhibitor cocktail	Sigma-Aldrich	
L-carnitine	Sigma-Aldrich	
[^{14}C]-acetate	PerkinElmer	NEC553050UC
[9,10-^{3}H(N)]-palmitic acid	PerkinElmer	NET043001MC
[9,10-^{3}H(N)]-oleic Acid	PerkinElmer	NET289001MC
[2,2',3,3'-^{3}H] octanoic acid	American Radiolabeling Chemicals	ART-1295-1
**Critical Commercial Assays**		
Free Glycerol Determination Kit	Sigma-Aldrich	FG0100
NEFA Reagent (NEFA-HR(2)	FUJIFILM Wako Diagnostics	
XF Cell Mito Stress Test Kit	Agilent	
Gentra Puregene Kits	Qiagen	
BCA protein assay	Thermo Fisher Scientific	
Phos-Tag gel	FUJIFILM Wako Chemicals	
**Experimental Models: Cell Lines**		
Mouse: mesenchymal stem cell	C57BL/6J	The Jackson Laboratory
Mouse: mesenchymal stem cell UCP1 KO	Ucp1^{tm1Kz}/J	The Jackson Laboratory
Mouse: mesenchymal stem cell ChREBP KO	kindly gifted by Dr. Lei Yin at the University of Michigan	

mouse stromal vascular cells	C57BL/6J	
Human white preadipocytes	kindly provided by Dr. Shingo Kajimura, Univerity of California San Francisco	
