# Supplementary material for: The molecular and metabolic program by which white adipocytes adapt to cool physiologic temperatures
Source: PLoS Biol. 2021 May 12;19(5):e3000988. doi: 10.1371/journal.pbio.3000988 (PMC8143427; doi:10.1371/journal.pbio.3000988)
Supplement: S1 Raw Images — (PDF) [file pbio.3000988.s016.pdf]

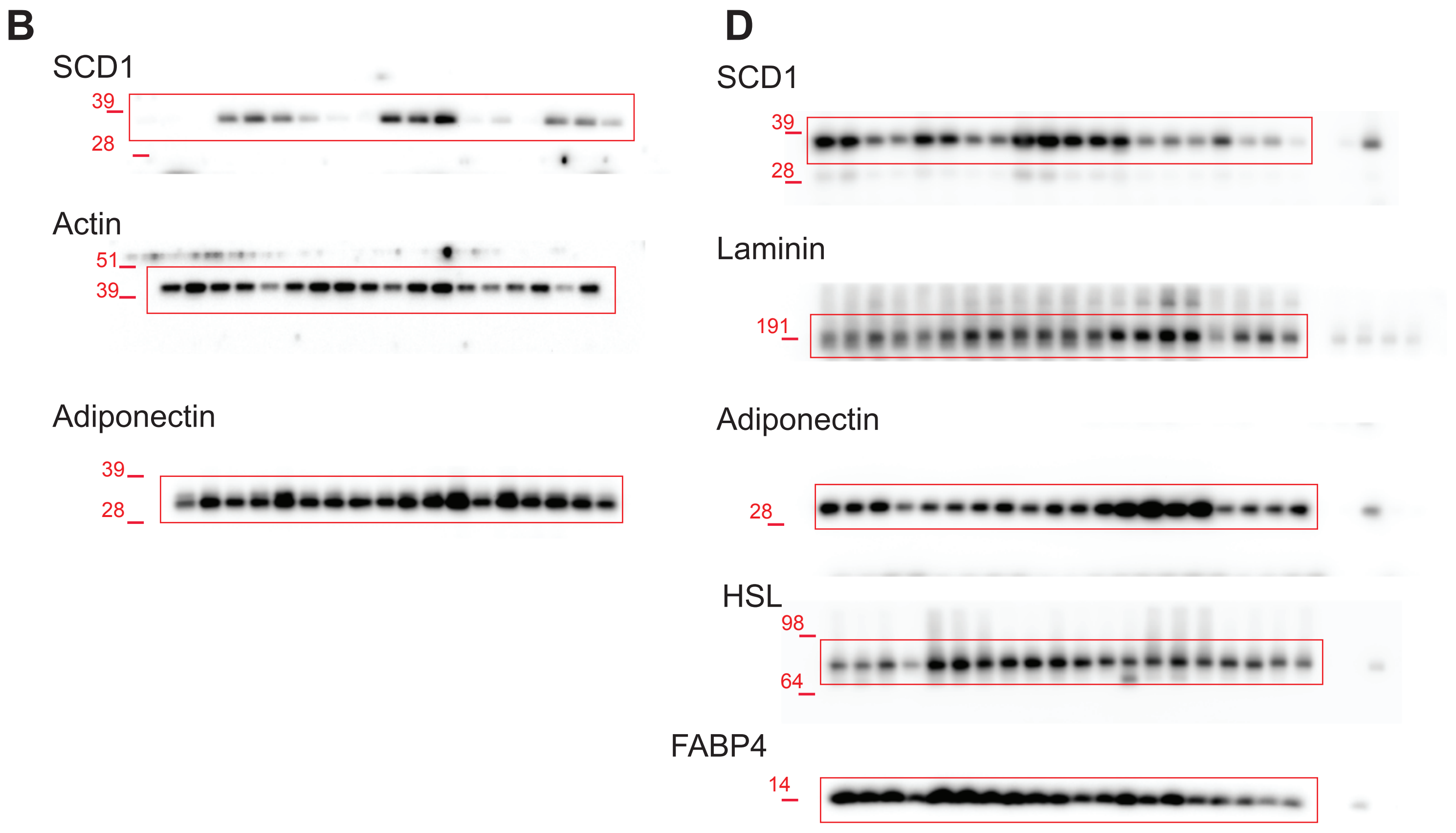

Original data for Figure 1

B

reflect vertically in order to prevent strong band and weak one are next each other

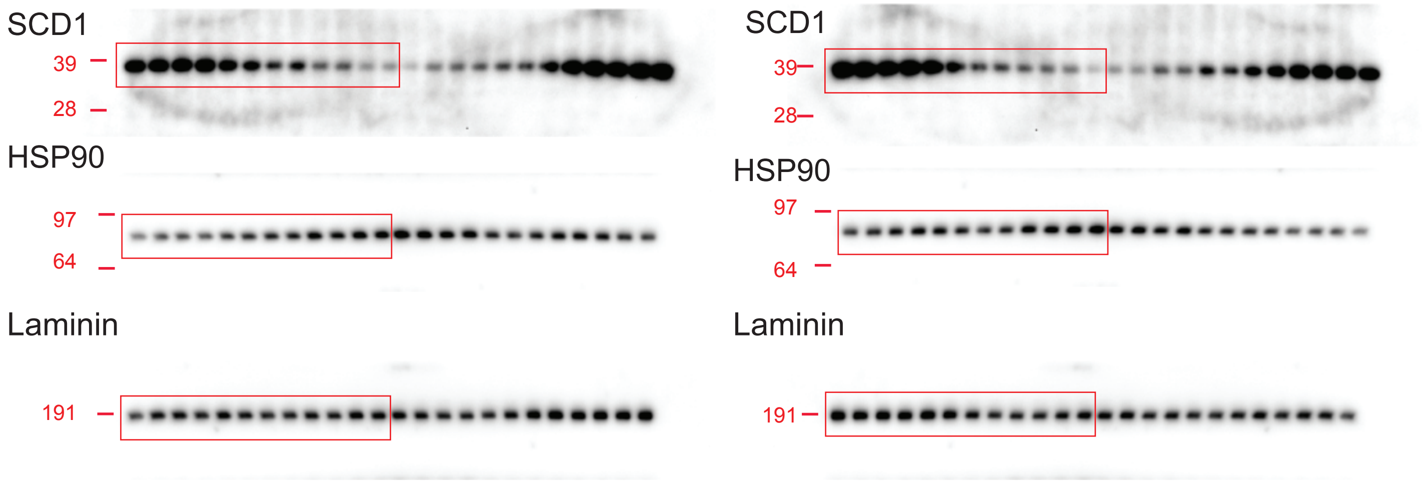

D

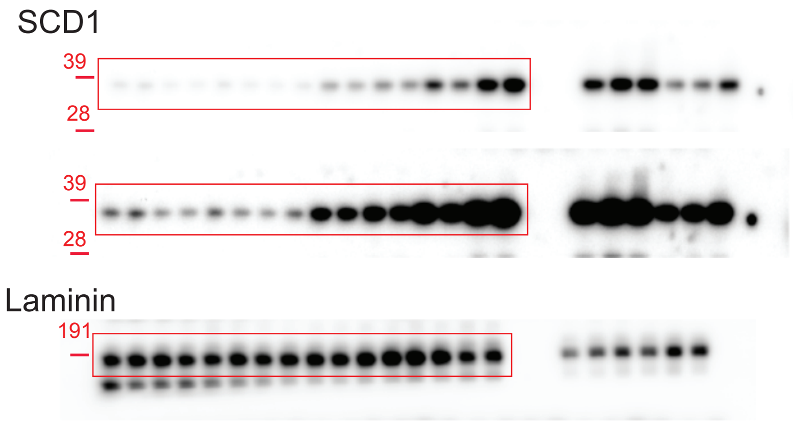

Original data for Figure 2

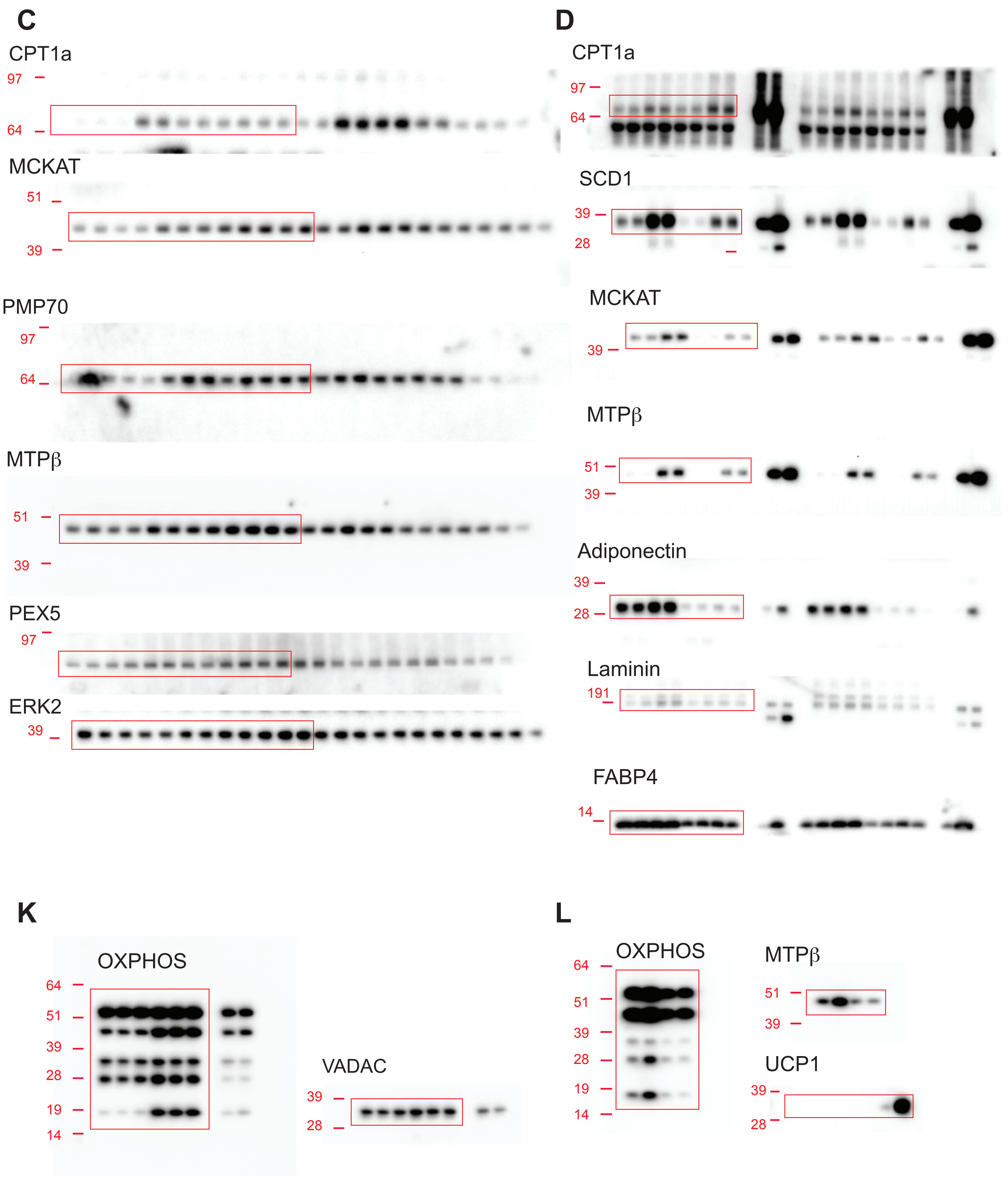

Original data for Figure 5

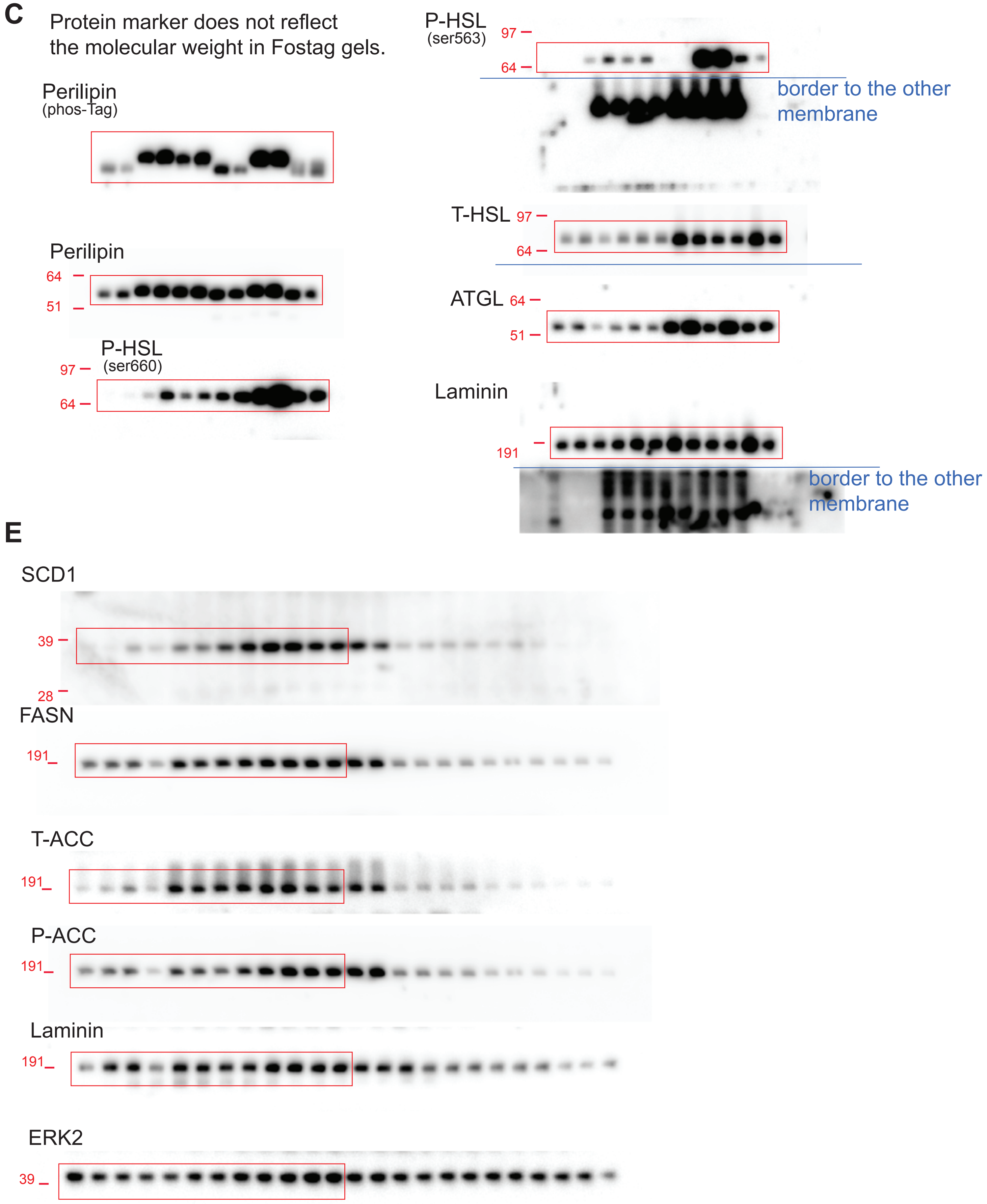

Original data for Figure 6

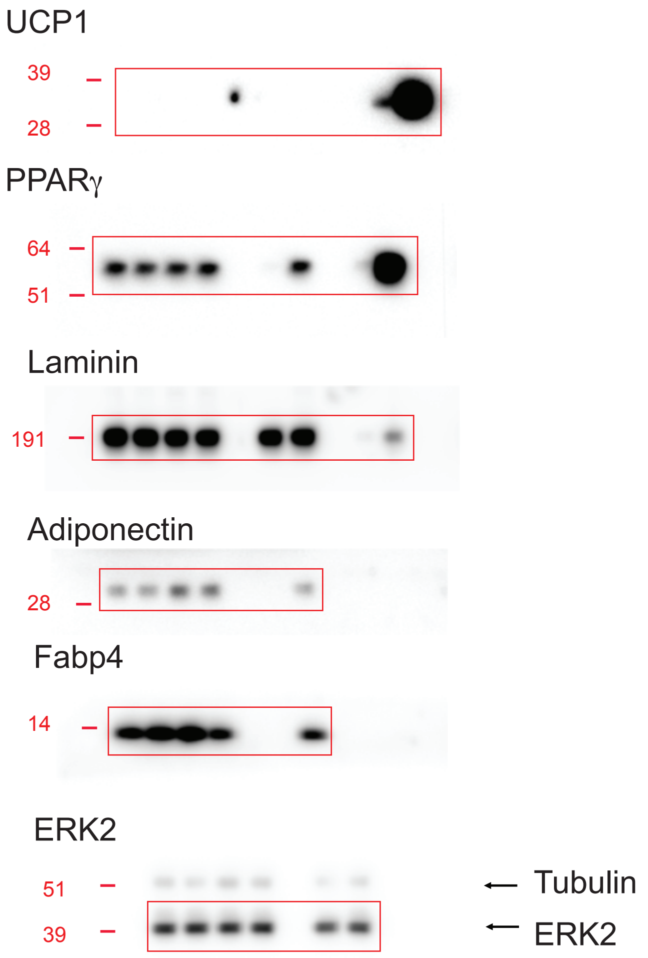

Original data for Figure S2

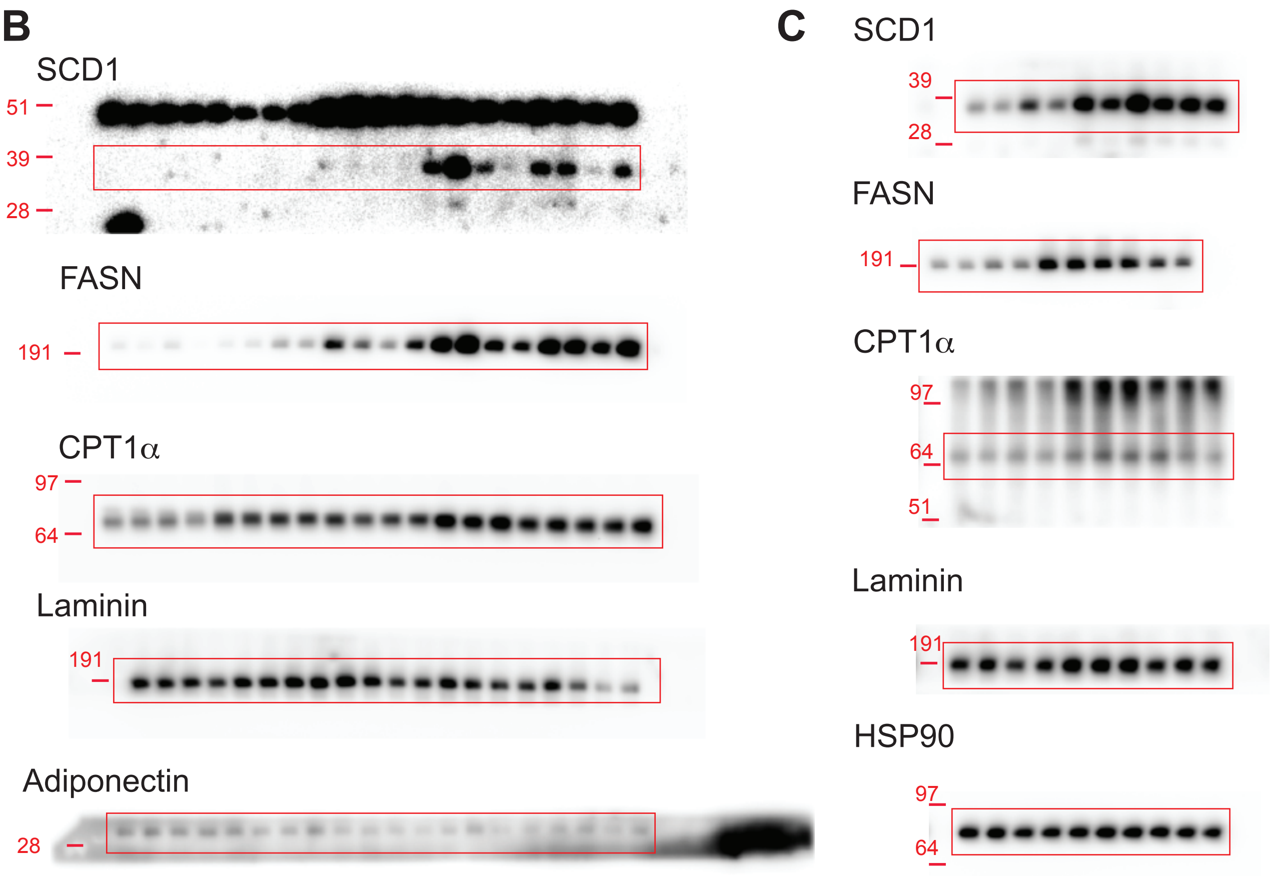

Original data for Figure S4

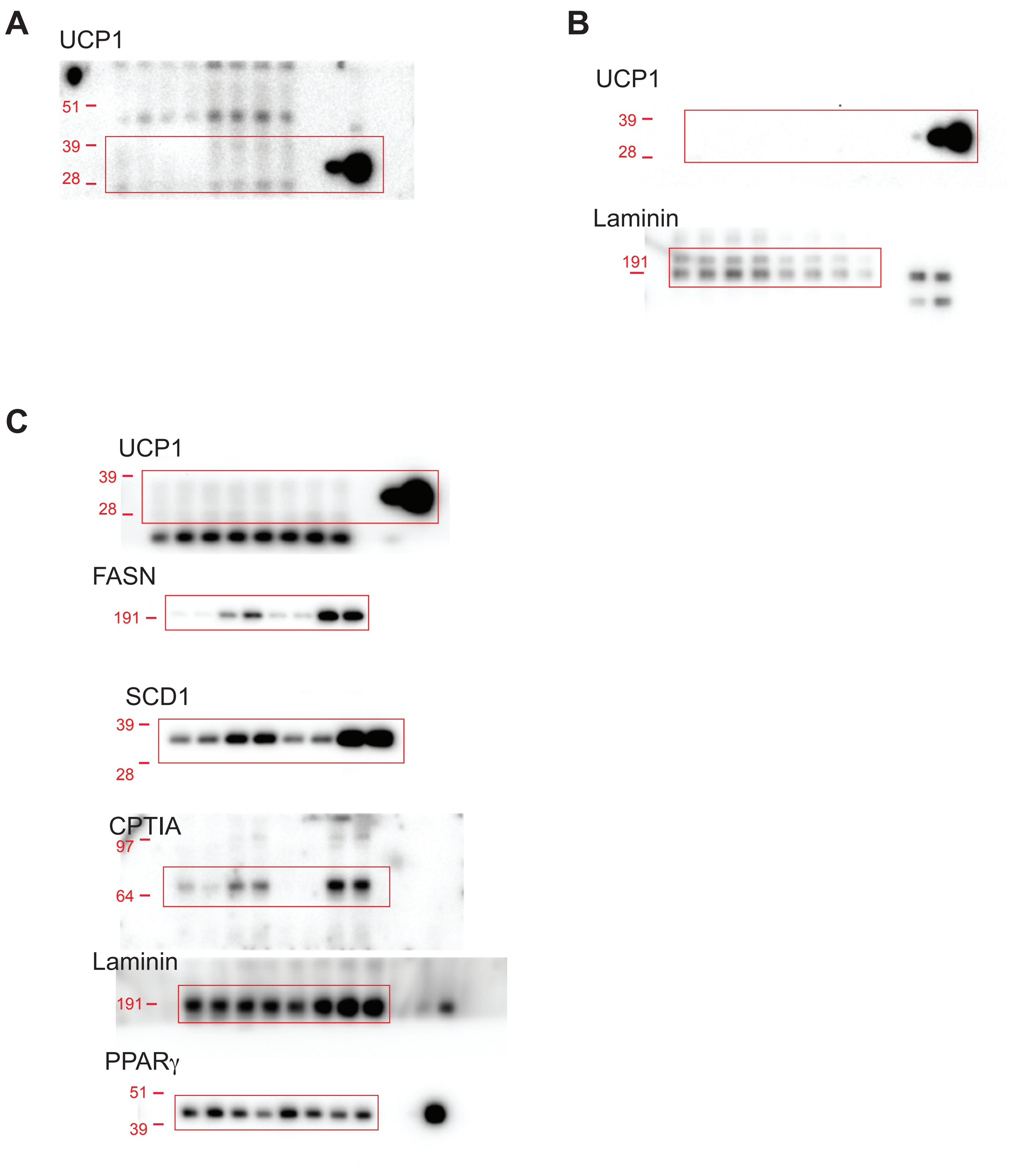

Original data for Figure S5

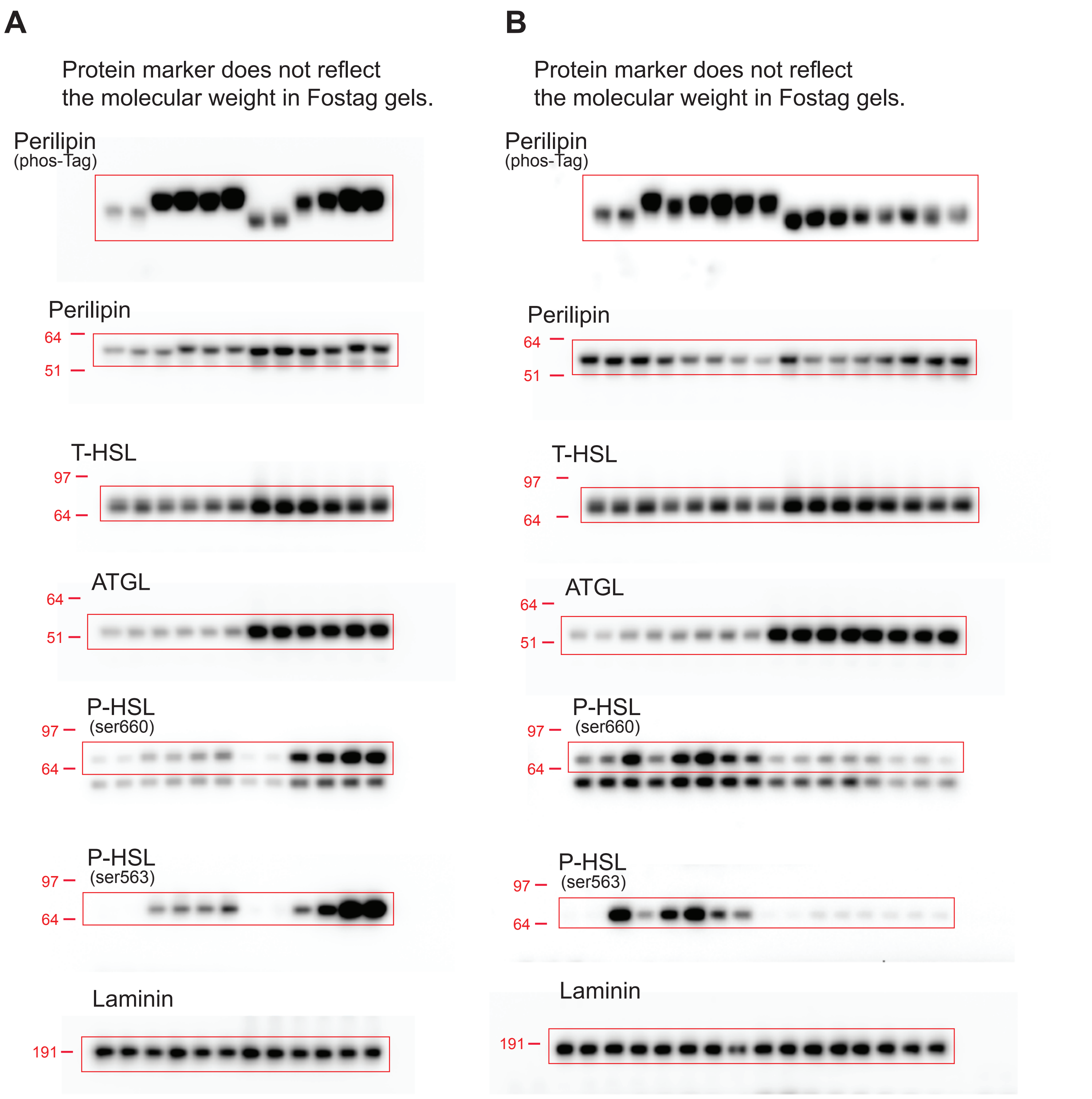

Original data for Figure S6

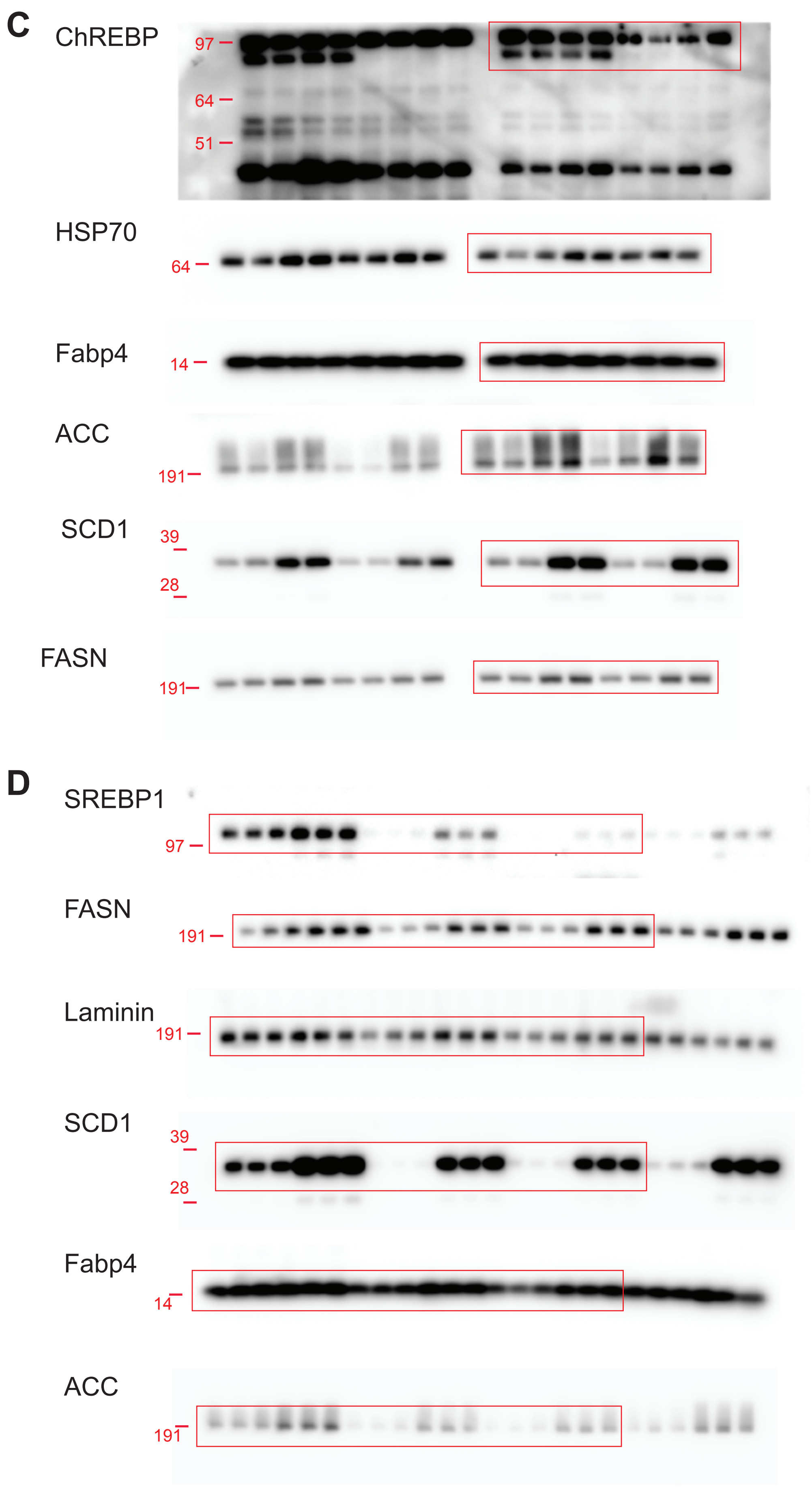

Original data for Figure S6
